# Brain region-specific disruption of *Shank3* in mice reveals a dissociation for cortical and striatal circuits in autism-related behaviors

**DOI:** 10.1038/s41398-018-0142-6

**Published:** 2018-04-27

**Authors:** Alexandra L. Bey, Xiaoming Wang, Haidun Yan, Namsoo Kim, Rebecca L. Passman, Yilin Yang, Xinyu Cao, Aaron J. Towers, Samuel W. Hulbert, Lara J. Duffney, Erin Gaidis, Ramona M. Rodriguiz, William C. Wetsel, Henry H. Yin, Yong-hui Jiang

**Affiliations:** 1 Departments of Neurobiology, Duke University, Durham, NC 27710 USA; 2 Pediatrics, Duke University, Durham, NC 27710 USA; 3 Psychology and Neuroscience, Duke University, Durham, NC 27710 USA; 4 Biology, Duke University, Durham, NC 27710 USA; 5 Genomics and Genetics Graduate Program, Duke University, Durham, NC 27710 USA; 6 Psychiatry and Behavioral Sciences, Duke University, Durham, NC 27710 USA; 7 Cell Biology, Duke University, Durham, NC 27710 USA; 8 Duke Institute for Brain Sciences, Duke University, Durham, NC 27710 USA

## Abstract

We previously reported a new line of *Shank3* mutant mice which led to a complete loss of *Shank3* by deleting exons 4−22 (Δe4−22) globally. *Δe4−22* mice display robust ASD-like behaviors including impaired social interaction and communication, increased stereotypical behavior and excessive grooming, and a profound deficit in instrumental learning. However, the anatomical and neural circuitry underlying these behaviors are unknown. We generated mice with *Shank3* selectively deleted in forebrain, striatum, and striatal D1 and D2 cells. These mice were used to interrogate the circuit/brain-region and cell-type specific role of *Shank3* in the expression of autism-related behaviors. Whole-cell patch recording and biochemical analyses were used to study the synaptic function and molecular changes in specific brain regions. We found perseverative exploratory behaviors in mice with deletion of *Shank3* in striatal inhibitory neurons. Conversely, self-grooming induced lesions were observed in mice with deletion of *Shank3* in excitatory neurons of forebrain. However, social, communicative, and instrumental learning behaviors were largely unaffected in these mice, unlike what is seen in global *Δe4*−*22* mice. We discovered unique patterns of change for the biochemical and electrophysiological findings in respective brain regions that reflect the complex nature of transcriptional regulation of *Shank3*. Reductions in Homer1b/c and membrane hyper-excitability were observed in striatal loss of *Shank3*. By comparison, *Shank3* deletion in hippocampal neurons resulted in increased NMDAR-currents and GluN2B-containing NMDARs. These results together suggest that Shank3 may differentially regulate neural circuits that control behavior. Our study supports a dissociation of Shank3 functions in cortical and striatal neurons in ASD-related behaviors, and it illustrates the complexity of neural circuit mechanisms underlying these behaviors.

## Introduction

Despite significant advances in identifying genetic defects in patients diagnosed with autism spectrum disorder (ASD), the anatomical basis and underlying neural circuit mechanisms that contribute to its core symptoms remain elusive^1,2^. These limitations represent a critical gap in our understanding of the disorder and hinder our ability to develop therapies targeting specific molecular or neural circuit abnormalities that underlie the condition. Human imaging studies of individuals affected by ASD have identified a pattern of morphological changes affecting many brain regions including the frontal cortex, hippocampus, amygdala, and striatum^3,4^. These clinical studies have suggested that local hyper-connectivity and long-range hypo-connectivity in forebrain structures may underlie the pathogenesis of ASD^5,6^. Early changes in neural circuit development and plasticity can result in lifelong impairments in the neural systems that subserve the core features of ASD^7^.

In particular, there are a number of reports implicating corticostriatal circuits in ASD^8–14^. Support for their role in the expression of ASD-associated behaviors is derived, in part, from neuroimaging studies comparing neurotypical and ASD subjects. Aberrant striatal morphology and growth trajectories in ASD subjects have been identified by MRI^4,15–17^, with perturbations in functional connectivity between the prefrontal cortex and basal ganglia^18–20^. While several studies have found correlations between corticostriatal imaging phenotypes and repetitive behaviors^15,21^, limitations in technique, heterogeneity of patient populations, and inability to perform direct manipulations limit our ability to demonstrate causality between the anatomical and behavioral manifestations of the disorder.

As a complement to human studies, experiments utilizing rodents provide a more mechanistic way to evaluate the role of specific neural circuits in the expression of ASD-like behaviors. Neural projections between the amygdala and hippocampus, as well as those between the ventral tegmental area and nucleus accumbens have been identified, and stimulation of these projections alter sociability^22,23^. With respect to other core ASD domains, the basal ganglia are hypothesized to contribute to repetitive behaviors, which are thought to involve aberrant striatal-mediated learning^24,25^. Recent studies, using optogenetics to target the orbitofrontal cortex to ventral striatum circuit, have found altering activity between these brain regions can induce or alleviate repetitive self-grooming^26,27^. However, most neural circuit studies in rodents have not been conducted in genetically-engineered mouse models with sufficient construct or face validity for ASD.

The *SHANK* genes (*SHANK1-3*) encode critical scaffolding proteins for glutamatergic neurotransmission in the post-synaptic densities (PSD) of neurons. Autism-causing mutations have been identified in all three *SHANK* genes^28^. Nevertheless, most mutations are found in *SHANK3*, which accounts for 1–2% of all ASD cases. Moreover, patients with a deletion containing *SHANK3* present with a high penetrance of ASD features^28–30^. *SHANK3* displays a complex transcriptional regulation that is cell type and developmental stage specific in brain due to the combination of multiple intragenic promoters and extensive splicing of coding exons^31^. Interesting to note, for the small number of cases carrying single nucleotide variants (SNVs) in *SHANK3*, the genetically deleterious mutations such as frameshift and nonsense mutations are exclusively localized in exon 21 and the rest of SNVs are missense in other exons^32^. To examine the role of *Shank3* in ASD-like behaviors, thirteen lines of *Shank3* isoform-specific mutant mice have been generated. These mice bear point mutations or deletions in various exons [∆e4−7, Δe4−9 (three lines), Δe9, Δe11, Δe13−16, e13−16^flex^, Δe21, e21^InsG3680^ (two lines), and e21^R1117X^]^33–43^. Despite these mouse models, these isoform-specific knockout lines have limited molecular construct validity as no patients with similar exonic deletions have been reported^32^ and only one ASD-pathogenic point mutation (InsG3680) has been identified within a single family^44^. We recently reported the first complete knockout of *Shank3* (*Δe4*−*22*), which recapitulates the mutations seen in the majority of patients with *SHANK3*-causing ASD^45^. The global *Δe4*−*22* mice display abnormal social behaviors, aberrant ultrasonic vocalizations (USVs), and increased repetitive responses that resemble the core behavioral features of the autism associated with *SHANK3*-related disorders. Hence, the *Δe4*−*22* line of mutant mice provides a unique opportunity to dissect the anatomical and neural circuit mechanisms underlying their ASD-like behaviors.

Relatively few studies have directly compared phenotypes using brain-region-specific mutant mice for ASD models^46,47^. Here, we report the molecular, physiological, and behavioral consequences of *Shank3 Δe4*−*22* deletions in specific corticostriatal regions using several different Cre drivers in transgenic mice. Our results reveal that *Shank3* deficiency in the neocortex is critical for the expression of increased grooming behaviors, while the striatum is critical for the expression of perseverative exploratory behaviors.

## Materials and methods

### Generation of *Shank3* mice with conditional deletion of exons 4−22

*Shank3 Δe4*−*22 mice* were generated using CMV-Cre to delete Shank3 in the germ-line^45^. *Drd1-Shank3* and *Drd2-Shank3* mice were generated by crossing the e4−22^flox/flox^ mice with dopamine (DA) D1 receptor (*Drd1*) Cre mice (B6.Cg-Tg (Drd1a-Cre)EY262Gsat/Mmcd) and DA D2 receptor (*Drd2*) Cre mice (B6.Cg-Tg(Drd2-Cre)ER44Gsat/Mmcd) [from The Gene Expression Nervous System Atlas (GENSAT) Project]^48^. *Dlx5/6-Shank3* mice were generated by crossing e4−22^flox/flox^ mice with Distaless5a/6a (*Dlx5/6*) Cre mice [Stock No. 008199; Jackson Laboratories, Bar Harbor, ME]^49^. *NEX-Shank3* mice were generated by crossing e4−22^flox/flox^ mice with NeuroD6 (*NEX*) Cre mice^50^. For each experiment, conditional knockout animals (Cre-positive, e4−22^flox/flox^) were compared to their own littermate control or “wild-type” animals (either Cre-negative e4−22^flox/flox^ or *Cre+*e4−22^+/+^). No differences were observed between these two genotypes from pilot data, so the genotypes were pooled into one control group for analysis. *Drd1a-*tdTomato mice were obtained from Dr. Nicole Calakos (Duke University, Durham, NC) and crossed to *Drd1-Shank3* and *Drd2-Shank3* mice for use in guiding the cell-type specific electrophysiological recordings. The natural *Disc1* mutation in 129/SvEv mice was segregated from the *Shank3* targeted mutation during the backcrossing^35^. See supplement for additional information on genotyping protocols and animal husbandry.

### Behavioral testing

Littermate WT and conditional *Shank3* KO mice (Supplemental Table 1) were tested in 6 cohorts of mixed sex (except for adult vocalizations, which were recorded only from male mice), with testing beginning at 8–12 weeks of age. All experimenters were blinded to genotype of the mice throughout the studies and the scoring of their behaviors, identifying animals by a subject number until the entire battery of tests was completed and analyzed at which point the genotypes were revealed. Many of the methods described below have been reported previously by our group^35,45,51,52^ and the details of testing are included in the Supplement. Behavioral testing was conducted with approved protocols from the Duke University Animal Care and Use Committee, which were in accordance with the NIH Guidelines for the Care and Use of Laboratory Animals^53^.

### Whole-cell patch clamp recording from brain slices

Recordings of action potentials were performed from medium spiny neurons (MSNs) in the dorsolateral striatal slices prepared from *Drd1-Shank3* and *Drd2-Shank3* mice crossed with *Drd1a-*tdTomato mice. After identifying direct pathway MSNs (D1) by the tdTomato signal, tdTomato-negative neurons were assumed to be indirect pathway MSNs (D2). Synaptic currents were recorded from hippocampal CA1 pyramidal neurons of *NEX-Shank3* mice. See Supplement for detail.

### Quantitative immunoblot analysis

Western blots were performed as previously reported by our group^45^. See supplement for detail.

### Statistical analyses

The data were analyzed with SPSS 21 (SPSS Inc., Chicago, IL) or Microsoft Excel and expressed as means ± SEM and analyzed by either two-tailed independent samples *t*-tests, analysis of variance (ANOVA), and repeated-measures ANOVA, depending on the number of groups and conditions of the experiment (see Supplementary statistics dataset). Sample sizes were based upon previous experience with similarly designed experiments or from pilot experiments.

## Results

### Generation of conditional *Shank3* knockout (KO) mice

**Fig. 1:**
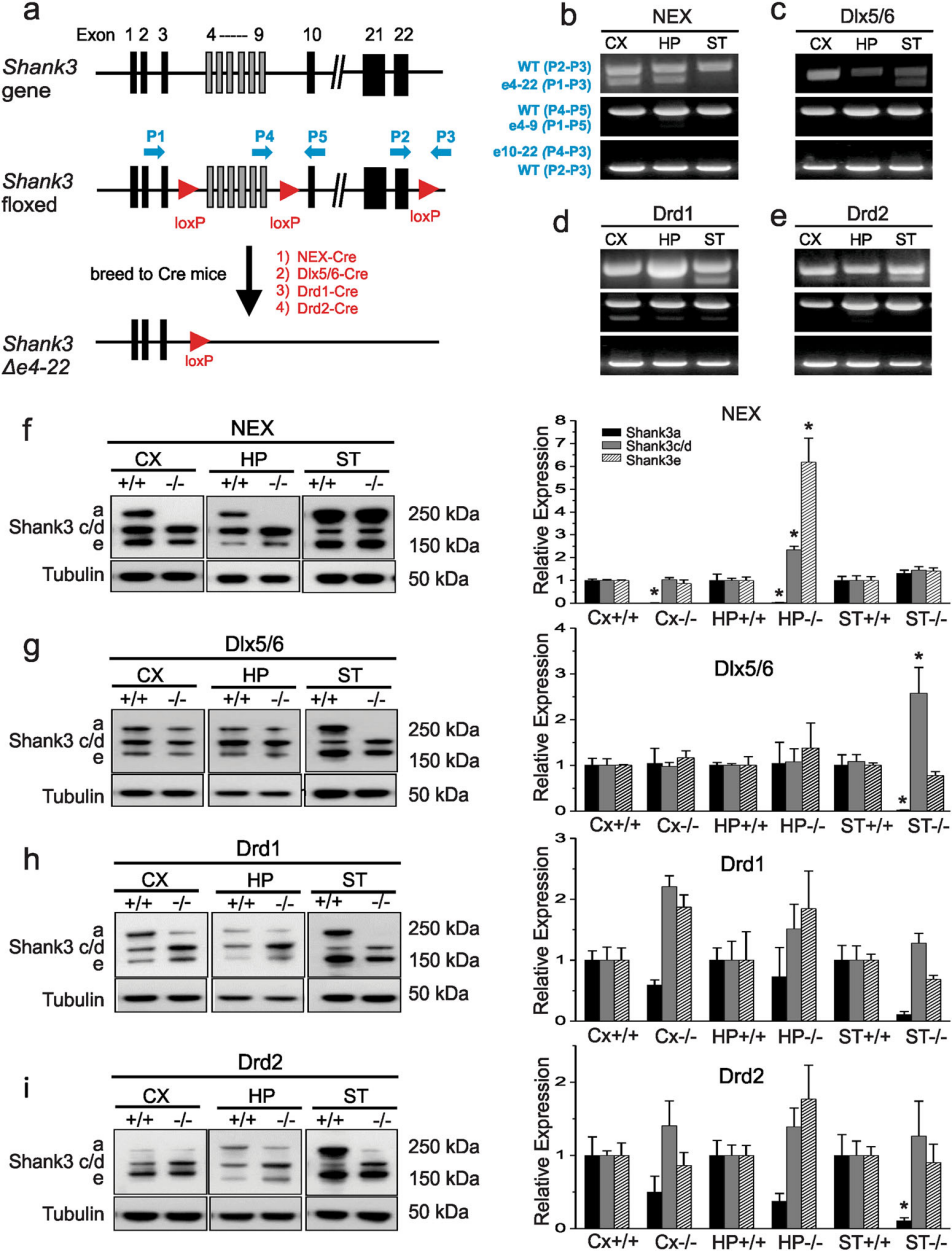
Conditional *Shank3 e4-22*^flox^ mice permit brain region-specific excision of *Shank3*. **a** The wild-type *mus Shank3* locus (*top*) depicting the engineered insertion of loxP sites (red arrowheads) before exon 4, after exon 9, and after exon 22 (*middle*). Crossing the *Shank3 e4–22*^flox/flox^ mice to Cre mice results in a two-step recombination ultimately at the first and third *loxP* sites, yielding deletion of Δe4–22 in Cre-expressing cells (*bottom*). Primers (blue arrows) are shown for detecting recombination of the *loxP* sites. **b–e** PCR-based detection of *Shank3* deletion of Δe4–22 (p1–p3), Δe4–9 (p1–p5), Δe10–22(p4–p3) in the cortex (CX), hippocampus (HP), and striatum (ST) of *NEX*-Cre *Shank3* floxed mice (*NEX*) (**b**), *Dlx5/6*-Cre *Shank3* floxed mice (*Dlx5/6*) (**c**), *Drd1*-Cre *Shank3* floxed mice (*Drd1*) (**d**), and *Drd2*-Cre *Shank3* floxed mice (*Drd2*) (**e**). A prominent deletion of e4–22 was observed in brain regions where corresponding Cres are predominantly expressed. **f** Western blots of dissected brains from *NEX-Shank3* mice reveals a loss of Shank3a protein in CX and HP, but not in ST from crude PSD fractions (two-way ANOVA, main effects of genotype and region and interaction, *p* ≤ 0.0001); paradoxically, Shank3c/d and Shank3e were increased in the HP (two-way ANOVA, main effects of genotype and region and interaction, *p* ≤ 0.002); *n* = 5/region/genotype. **g** Western blotting of dissected brains from *Dlx5/6-Shank3* mice reveals loss of Shank3a protein in the ST but not in the CX or HP crude PSD fractions (two-way ANOVA, main effects of region and interaction, *p* ≤ 0.05); with a similar paradoxical increase in Shank3c/d in the ST (two-way ANOVA, main effects of genotype and region and interaction, *p* ≤ 0.04) but no significant change for Shank3e; *n* = 5/region/genotype. **h** Western blotting of dissected brains of *Drd1-Shank3* mice reveals a loss of Shank3a protein in the ST, but not in the CX or HP crude PSD fractions (two-way ANOVA, main effect of genotype, *p*-value ≤ 0.02), although this did not withstand Bonferroni-corrected post-hoc comparisons and no significant differences were seen for Shank3c/d or Shank3e; *n* = 5/region/genotype. **i** Western blotting of dissected brains of *Drd2-Shank3* mice reveals loss of Shank3a protein in the ST but not in the CX or HP crude PSD fractions (two-way ANOVA, main effect of genotype, *p*-value ≤ 0.01), with no significant differences seen for Shank3c/d or Shank3e; *n* = 5/region/genotype. **f**–**i**, *p < 0.05, compared to the*+*/*+*control. All data are expressed as means ± SEM and were analyzed by two-way ANOVAs with genotype and brain region as factors; Bonferroni-corrected post-hoc comparisons

Since it has been hypothesized that cortico-striatal circuits underlie ASD-like behaviors, we crossed the recently generated transgenic mouse with loxP sites flanking *Shank3* exons 4−22 (e4−22^flox/flox^) to mice expressing Cre recombinase to disrupt the expression of Shank3 in cortical or striatal regions (Fig. 1a). We deleted *Shank3* in forebrain excitatory neurons of the cortex and hippocampus by crossing e4−22^flox/flox^ mice with NEX-Cre mice^50^ to generate *NEX-Shank3* mice, which begins to be expressed around embryonic day 11.5 (E11.5). To examine GABAergic neurons, and striatal MSNs in particular, we used Dlx5/6-Cre mice^49^ to produce *Dlx5/6-Shank3* mice, as it has been shown to be expressed in an enriched manner in GABA-ergic progenitors at similar timepoint (E12)^54^ which give rise to striatal MSNs and with relatively restricted robust expression in the striatum^55^. Numerous groups^55–58^ have used it to generate striatal-targeted conditional knockout lines of mice with robust striatal targeting, but not complete specificity, and with minimal effects in Cre^*+*^ control animals (lacking the floxed gene of interest)^59^. We also developed two additional lines of mice by crossing the e4−22^flox/flox^ mice with Drd1-Cre or Drd2-Cre mice^48^; thereby, selectively targeting the respective direct and indirect pathway MSNs of the basal ganglia. Parenthetically, expression of the specific lines of Drd1-Cre (EY262) and Drd2-Cre (ER44) begins on day E16 (Drd1-Cre) and day E18 (Drd2-Cre), respectively, and are not 100% restricted to the striatum, similar to the endogenous expression of *Drd1* and *Drd2*^48–50^. Using primers designed to detect recombination between exons 4 and 22 of the *Shank3* gene (Fig. 1a), we are able to detect the loss of *Shank3* in the cortex and hippocampus of homozygous floxed mice expressing NEX-Cre (*NEX-Shank3*) (Fig. 1b). Likewise, loss of *Shank3* exons 4−22 could be identified in striatal DNA samples from homozygous floxed mice expressing Dlx5/6-Cre, Drd1-Cre, and Drd2-Cre (*Dlx5/6-Shank3, Drd1-Shank3, Drd2-Shank3*, respectively) (Fig. 1c–e). To quantify the extent to which *Shank3* was deleted, we performed real-time PCR of genomic DNA and found that 25–50% of *Shank3* exons 4–22 were deleted in the hippocampus and cortex, respectively, of *NEX-Shank3* mice; 50% of *Shank3* was deleted in *Dlx5/6-Shank3* striatum; and 20–25% of *Shank3* was deleted in *Drd1-Shank3* and *Drd2-Shank3* striata (Supplemental Figure S1a–d). We were not able to design primers technically for qPCR to quantify the recombination between exon 4–9 and exon 10–22 due to the long genomic distance. Using three primers for PCR in the same reaction, we were able to assess the ratio of recombination between exon 4–9 and exon 10–22 semi-quantitatively. As shown in the Fig. 1b–e, we detected a low percentage of exon 4–9 deletion in Cre targeted tissues. However, the exons 10–22 deletion was not detectable which suggest that the recombination did not occur or they may overlap with the exon 4–22 deletion. *Shank3* is a transcriptionally complex gene with multiple promoters and extensive alternative splicing of coding exons^35,31^. The exact number and repertoire of mRNA isoforms are predicted to be large and the expression of known isoforms is brain region and cell type as well as development specific^35^. However, the transcript structure for most isoforms and expression patterns are largely uncharacterized due to the large size of mRNAs and lack of isoform specific antibodies. In a previous study, we have shown that we are able to examine five major mRNA isoforms (*Shank3a-e*) at the mRNA level using isoform-specific primer design^31^. Using the same design, we performed quantitative reverse-transcription PCR (RT-PCR) on RNA samples harvested from dissected brain regions of the four lines of conditional knockout mice (Supplementary Figure S1e–h). In all four lines of conditional KO mice, the full length *Shank3a* was almost completely disrupted in the tissues as expected. However, the reduction of other queried isoforms varied among different lines. For instance, in *NEX-Shank3* mice, *Shank3b* but not *Shank3 c-e* was significantly reduced in cortex (Supplementary Figure S1e). In *Dlx5/6*-*Shank3* mice, *Shank3b* in striatum and *Shank3d* in striatum, cortex, and hippocampus were affected significantly (Supplementary Figure S1f). In *Drd1-Shank3* mice, *Shank3b*, and *Shank3d* were reduced in striatum but *Shank3a* and *Shank3d* were also reduced (Supplementary Figure S1g). Lastly, in *Drd2-Shank3* mice, *Shank3b, c, d* were significantly reduced and *Shank3e* showed a trend of reduction in striatum (Supplementary Figure S1h).

Reduction of Shank3 protein in the expected brain regions was also confirmed with crude PSD fractions isolated from the cortex, hippocampus, and striatum of all 4 conditional *Shank3* knockout lines. Like our analyses of *Shank3* mRNA, reduction in Shank3a protein was observed in the cortex and hippocampus of *NEX-Shank3* mice (Fig. 1f) as well as in the striata of *Dlx5/6-Shank3, Drd1-Shank3*, and *Drd2-Shank3* mice (Fig. 1g–i). The reduction of the large molecular weight band in the western blot is consistent with the reduced expression of *Shank3a* from the qRT-PCR. However, we were not able to assess the isoform specific reduction at protein level because it is unknown how many Shank3 protein isoforms exist in different brain regions. As shown in Fig. 1f–i, some residual protein isoforms remained, suggesting either the efficiency of Cre mediated recombination was incomplete or likely, that other cell types such as astrocytes or other neuronal populations were not targeted by the Cre recombinase in a given brain region where the expression of *Shank3* isoforms has been described^31^. However, attempts to examine the isoform or cell type specific expression of *Shank3* in these lines of mutant mice were not successful due to lack of isoform specific and the inadequate quality for staining with Shank3 antibodies.

### *Shank3* conditional knockout mice engage in repetitive behaviors while social behaviors are intact

**Fig. 2:**
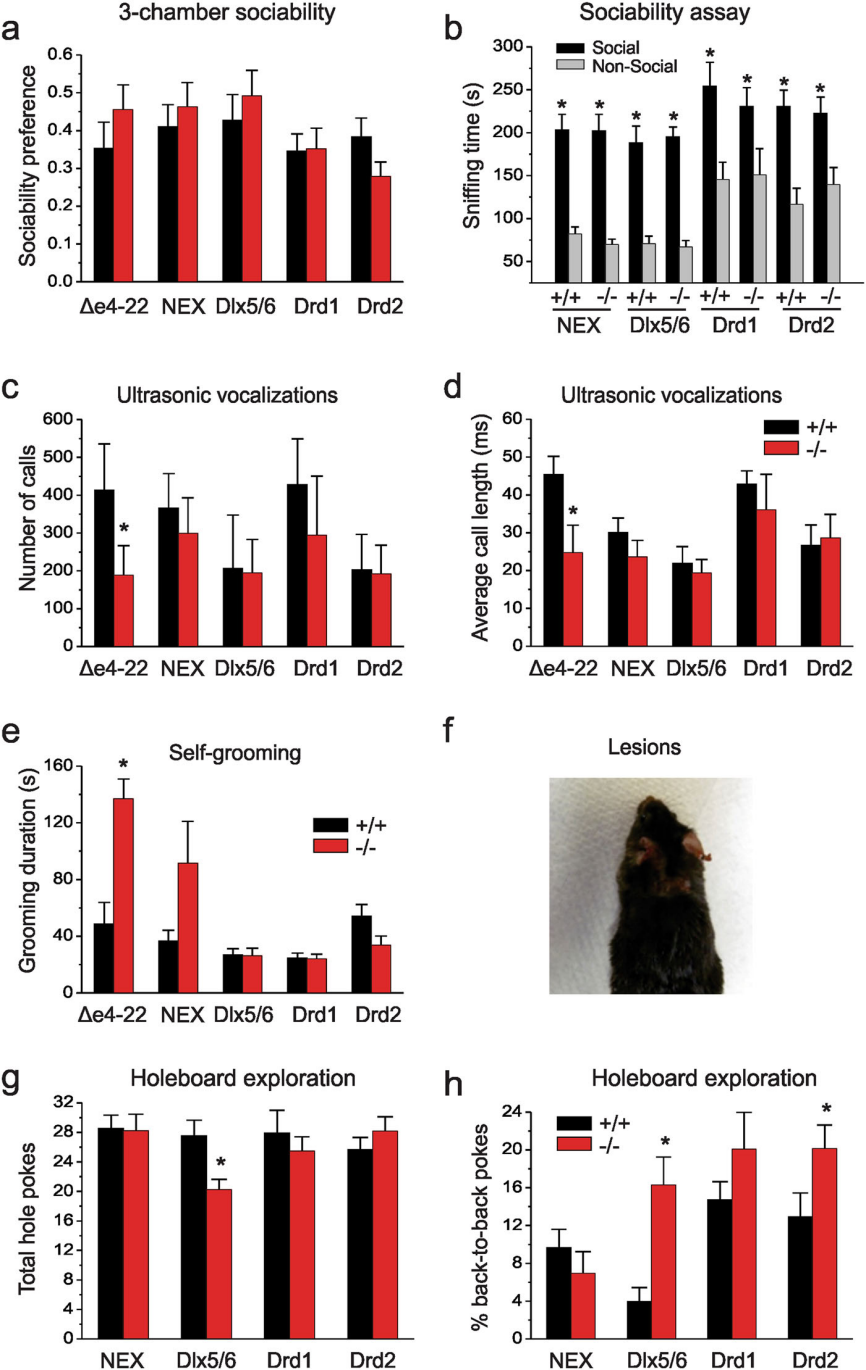
Repetitive behaviors persist in conditional *Shank3* knockout mice while social behavior and ultrasonic communication is intact. **a**, **b** Sociability assay. **a** All lines of mutant mice show normal social affiliation, preferring to interact with a novel mouse over that of an inanimate object when corrected for total time spent with either stimuli; t-tests, *n* = 9–16/genotype. **b** All lines showed significant increases in sniffing time of the social over the non-social stimuli (RMANOVA, main effects of stimulus, *p*-value ≤0.0001) but without genotype-related distinctions; *n* = 9–16/genotype. **c** While global *Δe4−22* mice emit fewer USVs (*p* = 0.002), all lines of conditional knockout mice (−/−) emit similar levels of calls as their wild-type controls (*+*/*+*); *n* = 7–16/genotype. **d** Only global *Δe4−22* mice emit USVs of shorter durations (*p* = 0.001) than their *+*/*+* littermates; *n* = 7–13/genotype. **e** Global *Shank3 Δe4−22* mice spend more time self-grooming (*p* = 0.0004), with a trend for *NEX-Shank3* mice (*p* = 0.086), whereas no genotype differences were found for any of the other lines of mutant mice; *n* = 11–18/genotype. **f** Approximately 25% (4/15) of the *NEX-Shank3* mice self-groomed to the point of producing self-injurious skin lesions, χ^2^(*n* = 30, df = 1) = 4.615, *p* = 0.032; *n* = 15/genotype. **g** In the hole-board test, *Dlx5/6-Shank3* knockout (−/−) mice made fewer nose-pokes (*p* ≤ 0.04) than their respective *+*/*+* controls, whereas no differences were seen in the other lines of mice; *t*-tests, *n* = 11–18/genotype. **h** *Dlx5*^*/*^*6-Shank3* (*p* = 0.001) and *Drd2-Shank3* mice (*p* = 0.050) made more repetitive nose pokes into single holes than *+*/*+* mice; neither *Drd1-Shank3* nor *NEX-Shank3* mice showed this tendency; *t*-tests, *n* = 11–18/genotype. For all panels, *p < 0.05, compared to the *+*/*+* control. All data are expressed as means ± SEM and were analyzed by independent samples two-tailed *t*-tests unless otherwise specified

We next evaluated cohorts of the WT littermates and conditional KO mice from each line for expression of core features of ASD-related behaviors. Mice were tested in 6 cohorts of mixed sex littermates by blinded observers. The detail statistical analysis for all behavioral tests are summarized in the Supplementary statistics dataset. The behaviors were not analyzed by sex, as we have not seen sex-specific differences in the behavioral phenotypes of *Δe4−22* mice (unpublished data) nor was this formally analyzed in our prior characterization of the global knockout^45^. In a test for social affiliation, we observed that the conditional KO mice from each line, as well as the global *Δe4−22* mice had no preference for either non-social stimulus (Supplemental Table 2). In testing, KO mice preferred interacting with the social over the non-social stimuli with levels of social affiliation similar to those of their WT littermates (Fig. 2a, b), indicating normal sociability. To examine social responses in a more naturalistic setting, we conducted the resident-intruder test in the *NEX-Shank3* and *Dlx5/6-Shank3* mice using a simplified ethogram based on our previous finding that non-reciprocated social approach is significantly increased in global *Δe4−22* mice^45^. However, we instead found a significant increase in bi-directional interactions between *Dlx5/6-Shank3* mice and the C3H intruders whereas the *NEX-Shank3* did not differ from their WT controls (Supplemental Table 2), but saw no significant differences in non-reciprocated interaction, the major social phenotype we observed in global *Δe4−22* mice^45^. We also examined ultrasonic vocalizations (USVs) in adult male mice exposed to estrus females. Unlike the global *Δe4−22* mice, there were no genotype differences in the numbers or durations of USVs in any line of the *Shank3* conditional KO mice (Fig. 2c, d).

As repetitive behaviors are evident in the global *Δe4−22* mice^45^, we monitored the duration of self-grooming in the home-cage of the different *Shank3* lines. We found that *NEX-Shank3* mice had a tendency for increased self-grooming but with significant variability (*p* = 0.086); however, altered self-grooming was not observed in *Dlx5/6-Shank3, Drd1-Shank3*, or *Drd2-Shank3* mice that targeted the basal ganglia for *Shank3* disruption (Fig. 2e). Furthermore, skin lesions similar to the global *Δe4−22* mice were observed in 4/15 *NEX-Shank3* mice compared to 0/15 in WT controls; notably, targeted deletion of *Shank3* in the striatum was insufficient to produce skin lesions in any of the three lines. This effect is consistent with an over-grooming phenotype similar to that in the global *Δe4−22* mice (Fig. 2f) with some *NEX-Shank3* engaging in very high levels of self-grooming similar to that of global *Δe4−22* mice. However, the penetrance was reduced (~25% of *NEX-Shank3* KOs developing skin lesions vs ~50% in global *Δe4−22* KOs) resulting in greater variability in the expression of this behavior.

We next examined another form of repetitive behavior as monitored in the hole-board test. While *Dlx5/6-Shank3* mice had significant reductions in the numbers of holes explored (Fig. 2g), perseverative or repetitive exploration of the same hole was augmented in *Dlx5/6-Shank3* and *Drd2-Shank3* mice (Fig. 2h). Together, these data suggest that loss of *Shank3* in forebrain excitatory neurons contributes significantly to the expression of repetitive self-grooming, whereas loss of *Shank3* in MSNs is responsible for the perseverative or repetitive exploration phenotypes seen in the global *Δe4−22* mice^45^.

### *Shank3* conditional knockout mice display distinctive comorbidities

We examined also multiple domains of learning which were reported to be abnormal in global *Δe4−22* mice^45^. To dissect the possible roles of the cortex-hippocampus and striatum to these responses, we focused on the *NEX-Shank3* and *Dlx5/6-Shank3* mice. Contextual fear has long been known to involve the hippocampus^60^. Although freezing behaviors were augmented in the global *Δe4−22* mice, both lines of the conditional KO mice demonstrated no genotype-dependent differences in freezing for contextual fear (Supplemental Figure S2a). Similarly, no genotype differences were observed for cued fear in any of the three genotypes (Supplemental Figure S2b). Given the profound deficits in instrumental learning seen in the global *Δe4−22* mice^45^ and the known role for the striatum in operant conditioning^24,25^, we utilized a lever-pressing task to determine whether deletion of *Shank3* in forebrain excitatory or basal ganglia inhibitory neurons was responsible for this phenotype. While global *Δe4−22* mice failed to acquire this task^45^, unexpectedly learning responses in both *Dlx5/6-Shank3* and *NEX-Shank3* mice were similar to that of their WT littermate controls (Supplemental Figure S2c, d).

**Fig. 3:**
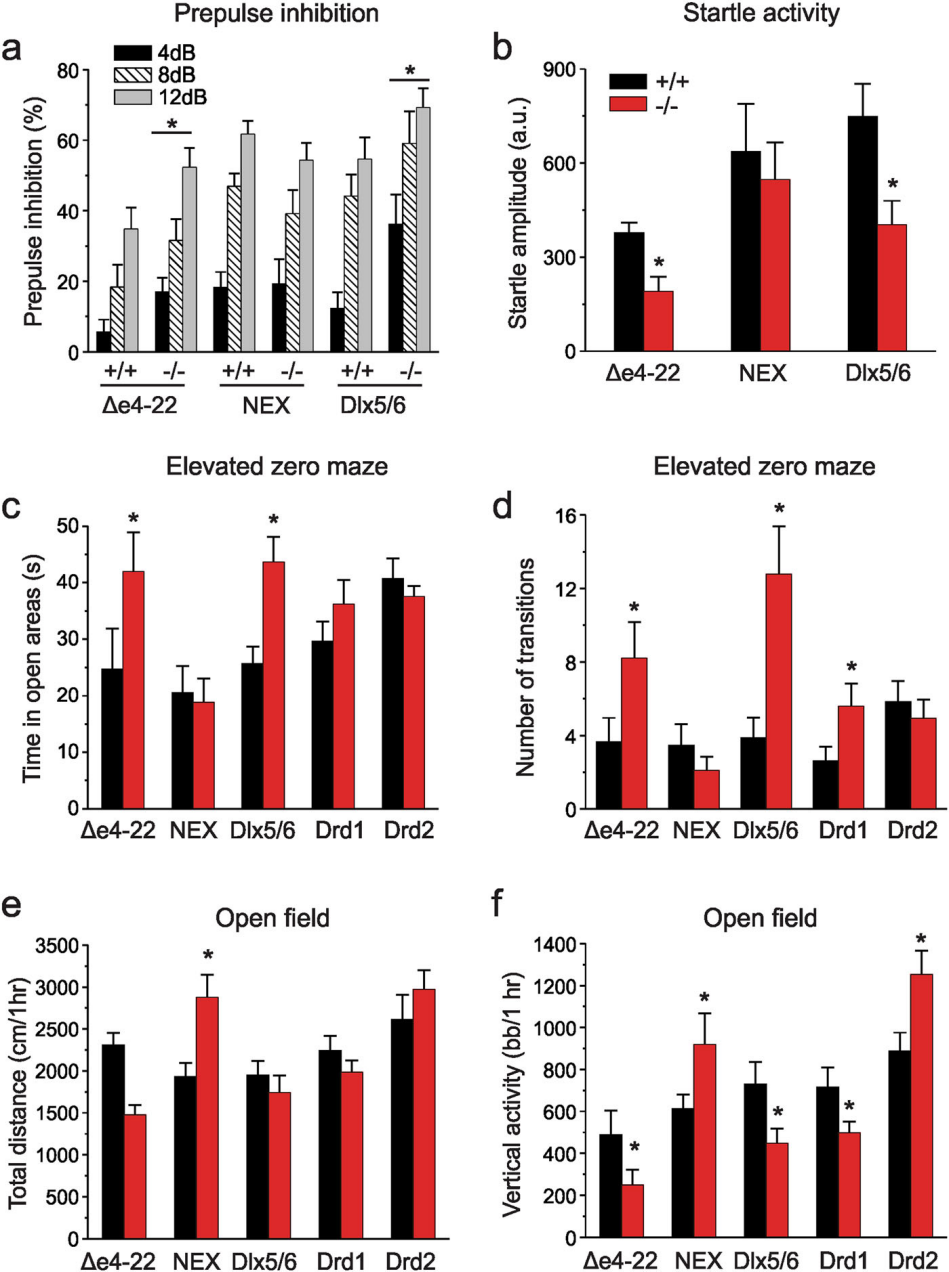
Distinctions among the *Shank3* conditional mice in anxiety-like behaviors and motor performance. **a** Prepulse inhibition (PPI) where genotypes within each strain were analyzed separately. While all mice showed increased PPI with increasing prepulse intensity (RMANOVA, main effect of intensity, *p* ≤ 0.001) global *Δe4−22* and *Dlx5/6-Shank3* mutant mice showed enhanced PPI across various intensities of prepulse stimuli relative to their *+*/*+* controls (main effect of genotype, *p* *≤* 0.05). No genotype differences were seen in *NEX-Shank3* mice; *n* = 9–12/genotype. **b** Startle activities in global *Δe4−22* and *Dlx5/6-Shank3* (*t*-tests, *p* ≤ 0.02) were reduced relative to their *+*/*+* littermates, whereas startle amplitudes in *NEX-Shank3* mice were similar to those of their *+*/*+* littermates; *n* = 9–12/genotype. **c**, **d** Elevated zero maze for anxiety-like behaviors. **c** Similar to the global *Δe4−22* mice*, Dlx5/6-Shank3* mice spend more time in the open areas of the maze than their *+*/*+* controls (*t*-tests, *p* ≤ 0.05); *n* = 9–18/genotype. Responses in the *NEX-Shank3*, *Drd1-Shank3, Drd2-Shank3* were similar to those of their *+*/*+* controls. **d** *Dlx5/6-Shank3* (*t*-test, *p* = 0.006) and *Drd1-Shank3* (*t*-test, *p* = 0.050) mice also make more transitions from the closed-to-open-to-closed areas, as is seen in global *Δe4−22* mice (*t*-test, *p* = 0.008); *n* = 9–19/genotype. **e**, **f** Open field activity. **e** Global *Δe4−22* mice traveled over a shorter distance in the open field (*t*-test, *p* = 0.056), whereas locomotion in *NEX-Shank3* mice was greater than that of their *+*/*+* littermates (*t*-test, *p* = 0.004). No significant differences in locomotion were seen in *Dlx5/6-Shank3, Drd1-Shank3*, or *Drd2-Shank3* mice; *n* = 12–18/genotype. **f** *Dlx5/6-Shank3* (*t*-test, *p* = 0.038) *Drd1-Shank3* (*t*-test, *p* = 0.050), and global *Δe4−22* mice (*t*-test, *p* = 0.010) mice all demonstrated lower rearing behavior. By contrast, *NEX-Shank3* (*t*-test, *p* = 0.025) and *Drd2-Shank3* mice (*t*-test, *p* = 0.015) demonstrated increased rearing; *n* = 12–18/genotype; bb/ 1 h = beam breaks in 1 h. For all panels, *p < 0.05, compared to wild-type controls. All data are expressed as means ± SEM and were analyzed by independent samples two-tailed *t*-tests unless otherwise specified

As patients with idiopathic and *SHANK*-related ASD and animal models of ASD often exhibit sensory abnormalities, including abnormalities of sensorimotor gating^61,62^, we examined responses in a prepulse inhibition (PPI) paradigm. Based on the role of the ventral striatum in the regulation of PPI^63^, we hypothesized that a reduction of Shank3 in the MSNs would alter PPI performance^64^. Indeed, we found that PPI was augmented in global *Δe4−22* and *Dlx5/6-Shank3* mice relative to their WT controls, whereas PPI in *NEX-Shank3* mice was similar to that of their WT littermates (Fig. 3a). However, startle activities were reduced in the global *Δe4−22* and *Dlx5/6-Shank3* mice (Fig. 3b) which complicates interpretation of the altered PPI response, although the recapitulation in *Dlx5/6-Shank3* mice of both findings observed in the *Δe4−22 mice* suggests that loss of Shank3 in striatal neurons may be responsible for the sensorimotor gating differences.

Given how strongly anxiety-like behavior, a frequent comorbidity in ASD patients including those with *SHANK*-related ASD, was affected in the global *Δe4−22* mice^45^, we hypothesized that this behavioral phenotype would be recapitulated in the NEX-Cre targeted mutants. Cre expression should disrupt *Shank3* in the amygdala and ventral hippocampus—key neural substrates for anxiety-like behavior^23,65,66^. We examined anxiety-like behavior in the elevated zero maze. Unlike their previously observed increase in anxiety-like behavior in the light-dark boxes^45^, global *Δe4-22* mice spent more time in the open areas of the maze and made more closed-to-open-to-closed arm transitions than their WT littermates (Fig. 3c). While the reason for this discrepancy is unclear, it was a robust phenomenon observed across multiple cohorts of mice in various unpublished studies. A similar phenotype was recapitulated in the *Dlx5/6-Shank3* mice. An examination of transitions between the closed-to-open-to-closed areas of the maze revealed that the global *Shank3 Δe4−22*, *Dlx5/6-Shank3*, and *Drd1-Shank3* mice engaged in more transitions than their WT littermate controls (Fig. 3d).

Locomotor activity was examined also in the global *Δe4−22* mice^45^. Since the basal ganglia is known to play a key role in modulating motor activity^25,67^, we hypothesized that locomotor activity would be affected by striatal deletion of *Shank3*. While global *Δe4−22* mice displayed a tendency towards hypoactivity in this study and were significantly hypoactive in prior testing^45^, the *NEX-Shank3* mice were hyperactive (Fig. 3e). By contrast, locomotion in the lines of striatal-targeted *Shank3* mutant mice did not differ from their WT controls. An examination of rearing activities revealed that this behavior was attenuated in global *Shank3 Δe4−22, Dlx5/6-Shank3*, and *Drd1-Shank3* mice, whereas it was increased in *NEX-Shank3* and *Drd2-Shank3* mice (Fig. 3f). This reduction in rearing did not appear to be due to anxiety-like behavior in this context, as all five KO lines spent similar amounts of time in the center of the open field compared to their littermate controls (Supplemental Figure S2e). Another deficiency reported in the global *Δe4−22* mice was their impairment on the rotarod^45^. We evaluated whether loss of Shank3 in the striatum would selectively affect their performance. On the accelerating rotarod, only a minor trial-specific reduction was observed in the *NEX-Shank3* mice (Supplemental Figure S2f), performance in the other lines of conditional KO mice was similar to that of their WT littermates (Supplemental Figures S2g–i).

### *Shank3* deletion in direct and indirect pathway MSNs exert cell-autonomous effects on neuronal excitability and reduces scaffolding to Homer1b/c

**Fig. 4:**
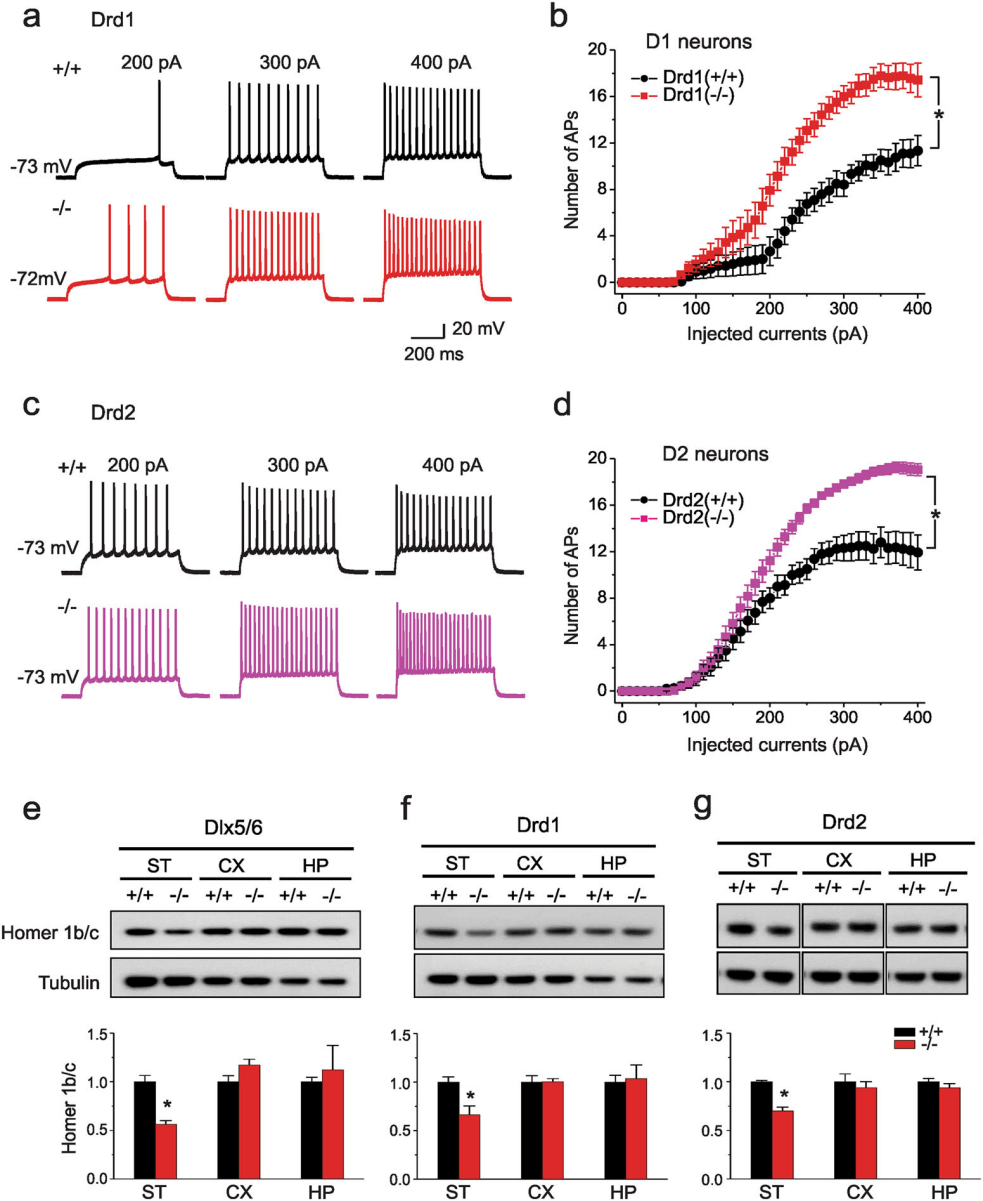
Loss of *Shank3* in selected striatal neurons leads to cell autonomous alterations of synaptic function and PSD components. (**a**) Representative traces of evoked action potentials in D1 MSNs neurons from *Drd1-Shank3* WT (*+*/*+*) (black) and KO (−/−) (red) mice. The action potentials reflect responses to 200, 300, and 400 pA current injections, respectively. **b** Summarized data for the number of evoked action potentials (APs) at the indicated amplitudes of current injection in D1 MSNs from *Drd1-Shank3* WT (*+*/*+*) and KO (−/−) mice (2-way ANOVA, main effects of genotype and stimulation, *p* < 0.001, genotype x stimulation interaction, *p* < 0.001). **c** Example traces of evoked action potentials in D2 MSNs neurons from *Drd2-Shank3* WT (*+*/*+*) (gray) and KO (−/−) (pink) mice. **d** Summarized data for the numbers of evoked action potentials at the indicated amplitudes of current injection in D2 MSNs from *Drd2-Shank3* WT (*+*/*+*) and KO (−/−) mice (2-way ANOVA, main effects of genotype and stimulation, *p* < 0.001, genotype x stimulation interaction, *p* < 0.001). **e**−**g** Homer1b/c levels in the PSD from striatum where loss of *Shank3* was targeted. (e) *Dlx5/6-Shank3* mice show a reduction in Homer1b/c protein in striatal (ST) (*p* = 0.002), but not in cortical (CX) or hippocampal (HP) PSD samples; *n* = 5 mice/genotype. **f** *Drd1-Shank3* mice have decreased Homer 1b/c in ST (*p* = 0.018), but not in the CX or HP samples; *n* = 4 mice/genotype. **g** *Drd2-Shank3* mice have a loss of Homer1b/c in the ST (*p* < 0.001), but not in the CX or HP; *n* = 4 mice/genotype. For all westerns, independent samples two-tailed *t*-tests; representative images are shown and each western was replicated at least two times. For all panels, *p < 0.05, compared to wild-type controls. All data are expressed as means ± SEM

Since we found that various behavioral phenotypes seen in the global *Δe4–22* mice were recapitulated by brain-region selective targeting of *Shank3* deletion, we next queried whether any of the electrophysiological and biochemical phenotypes found in the global *Δe4–22* mice could be dissociated using this conditional deletion approach^45^. Since many of these cellular and synaptic phenotypes were most prominent in the striatum, we examined whether loss of *Shank3* in direct or indirect pathway MSNs may account for these phenotypes. Using a transgenic reporter line of mice expressing tdTomato in Drd1-containing MSNs^68^, we performed patch-clamp recordings on putative Drd1 (tdTomato*+*) and Drd2 (tdTomato-) neurons from *Drd1-Shank3* and *Drd2-Shank3* mice and their respective WT controls (Supplemental Figure S3a). We examined excitability in these cells, as MSNs from global *Δe4−22* mice are hyper-excitable relative to WT controls^45^. Single action potentials were evoked by a 10-ms current injection in 5-pA increments to determine the current threshold to initiate an action potential. D1 cells from *Drd1-Shank3* mice had markedly decreased current thresholds for action potentials (Supplemental Figure S3b). We also analyzed the number of action potentials evoked over a wide range of current amplitudes. The numbers of action potentials evoked were significantly increased in D1 cells from *Drd1-Shank3* mice than from D1 cells of WT littermates (Fig. 4a, b). However, control D2 cells from *Drd1-Shank3* mice, in which *Shank3* was not deleted, had thresholds and excitability profiles that were indistinguishable from D2 cells from WT controls (Supplemental Figure S3d). The converse was observed in *Drd2-Shank3* mice, with D2 cells of the mutant animals being hyper-excitable relative to D2 cells of WT controls with increased action potential firing in response to current injection (Fig. 4c, d) although the threshold to action potential generation did not differ between genotypes (Supplemental Figure S3c). Control D1 cells from *Drd2-Shank3* mice, in which *Shank3* was not targeted, had responses similar to the D1 cells of their WT littermates (Supplemental Figure S3e). Other electrophysiological properties (Supplemental Table 4) such as input resistance, resting membrane potential, peak amplitude, and action potential kinetics were largely non-differentiated among genotypes. However, there was a significant increase in input resistance and significant reductions in the current threshold to initiate an action potential and a deploarized resting membrane potential in D1 neurons of *Drd1-Shank3* mice relative to the D1 neurons of their WT controls. Hence, the electrophysiological results indicate that *Shank3* is expressed in both D1 and D2 cells and its loss from either cell type is sufficient to alter the excitability of these neurons autonomously.

We next examined whether changes in the PSD scaffolds of these cells could account for some of the observed effects. We focused first on Homer1b/c, given that it is consistently observed to be diminished across multiple mouse models of *Shank3* deficiency^34,35,40,41,43^ and its protein level correlates with the degree of behavioral impairment in the global *Δe4−22*-mice^45^. Additionally, pharmacological manipulations of the metabotropic glutamate receptor mGluR5, which serves as a scaffold for Homer 1b/c and Shank3, can ameliorate some behavioral phenotypes in these mice^45^. Striatal PSD fractions from *Dlx5/6-Shank3* (Fig. 4e), *Drd1-Shank3* (Fig. 4f), and *Drd2-Shank3* (Fig. 4g) mice were found to have a significant reduction in Homer1b/c levels which were not observed in cortical or hippocampal samples from these three mouse lines.

### *Shank3* deletion in forebrain excitatory neurons increases NMDAR synaptic function and subunit protein levels

**Fig. 5:**
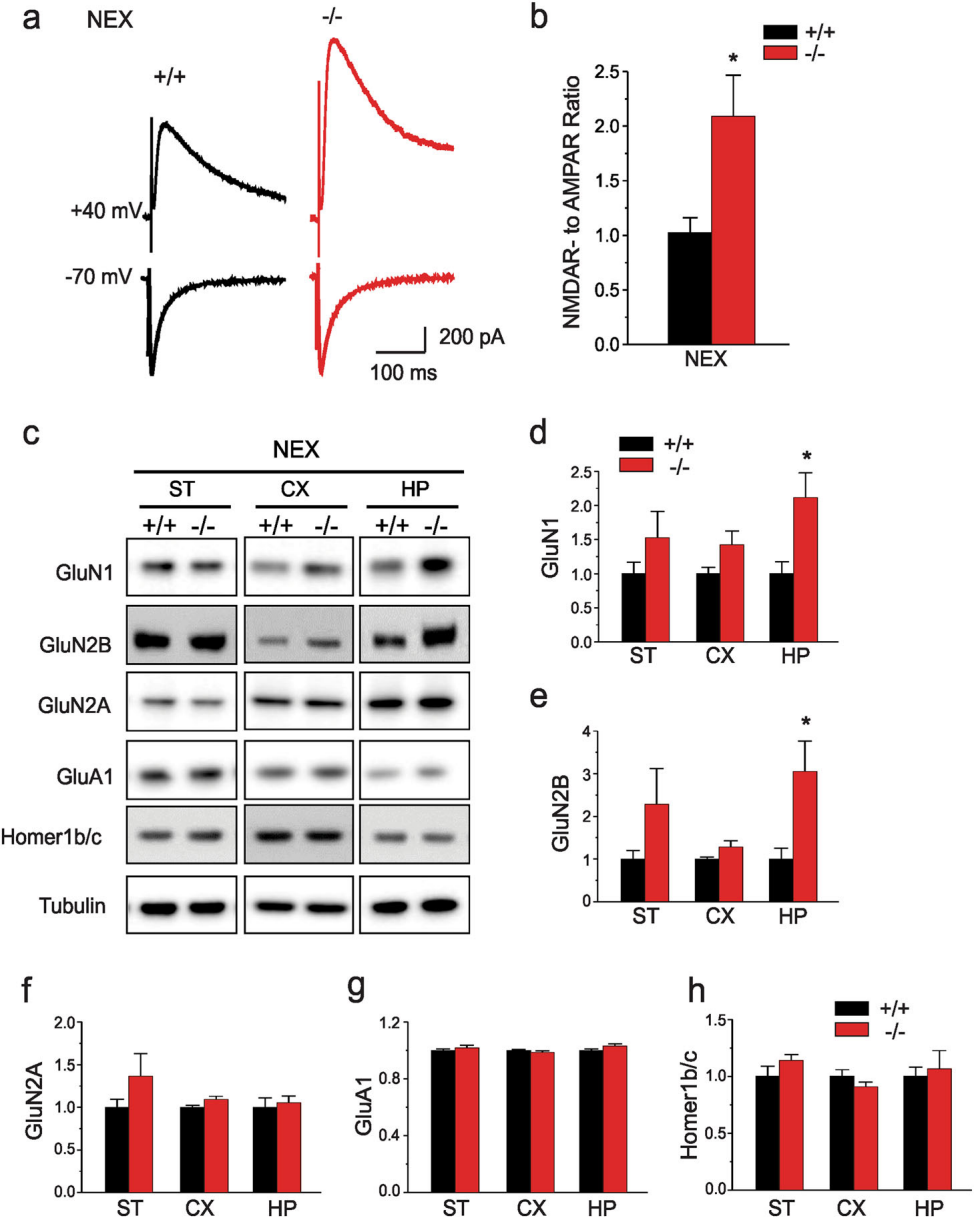
Loss of *Shank3* in hippocampal neurons leads to increased NMDA synaptic function and alterations in receptor subunits. **a** Representative traces of NMDAR-EPSCs and AMPAR-EPSCs recorded at the same stimulation intensities (300 µA) and in the same CA1 neurons from *NEX-Shank3* WT (*+*/*+*) and KO (−/−) mice. **b** Bar graph of the NMDAR- to AMPAR-EPSC ratio in *NEX-Shank3* WT (*+*/*+*) and KO (−/−) mice (*t*-test; *p = 0.009). **c** Immunoblotting of PSD components from striatum (ST), cortex (CX), and hippocampus (HP) of *NEX-Shank3* mice. **d** *NEX-Shank3* mice have increased GluN1 protein in the CX (*t*-test; *p* = 0.059) and HP (*t*-test; *p* = 0.019), but not in the ST. **e** Samples from these mice also have augmented GluN2B protein in the HP (*p* = 0.022), with a tendency for increased levels in the CX (*t*-test; *p* = 0.102), but not in the ST. **f**–**h** GluN2A (**f**), GluA1 (**g**), and Homer1b/c (**h**) did not show any significant changes between *NEX-Shank3* mice and WT littermates in any of the brain regions. *n* = 6 mice/genotype for all westerns; representative images shown and each western was replicated at least two times. For all panels, independent samples two-tailed t-tests; *p < 0.05, from *+*/*+*. All data are expressed as means ± SEM

Given the unique subset of behavioral phenotypes present in *NEX-Shank3* mice, we examined whether additional synaptic components may be dysregulated in these mice. As hippocampal circuitry is well-established and neuronal populations are more homogeneous in this structure than in cortical preparations, we recorded from hippocampal CA1 neurons of *NEX-Shank3* mice. For these studies, we examined the functions of AMPARs and NMDARs which are scaffolded by Shank3 in the PSD and have been found to be altered in the hippocampi of some lines of isoform-specific *Shank3* knockout mice^34–36,39,41^. First, we recorded NMDAR-mediated excitatory postsynaptic currents (EPSC_S_). Induction of NMDAR-EPSCs by a series of stimulus intensities was markedly enhanced in *NEX-Shank3* mice (Supplemental Figures S4a, b). In contrast, AMPAR-EPSCs were unchanged in CA1 pyramidal neurons from the *NEX-Shank3* mice compared to WT controls (Supplemental Fig. 4c, d). Additionally, the NMDAR- to AMPAR-EPSC ratio was significantly larger in CA1 pyramidal neurons from *NEX-Shank3* mice than those from WT controls (Fig. 5a, b).

To examine the possible biochemical basis for our electrophysiological findings, we performed quantitative immunoblots of crude PSD proteins isolated from dissected brain regions of *NEX-Shank3* mice and their WT littermates (Fig. 5c). Immunoblotting for the obligatory subunit of the NMDAR, GluN1 revealed a significant increase in *NEX-Shank3* hippocampus with a trend for an enhancement in the cortex (Fig. 5d). We observed similar results when blotting for GluN2B (Fig. 5e); however, no significant changes were observed in GluN2A levels (Fig. 5f). In agreement with our electrophysiological data, immunoblotting for the obligatory AMPAR subunit, GluA1, revealed that levels in the cortex and hippocampus were similar between WT and *NEX-Shank3* mice (Fig. 5g). Parenthetically, we failed to observe any significant changes in Homer1b/c protein in any of the *NEX-Shank3* samples (Fig. 5h). This was expected given the mild to moderate changes in Homer1b/c levels observed in the neocortex of global *Δe4−22* mice^45^.

## Discussion

Our analyses of selective *Shank3* deficiency in the forebrain and striatum have revealed several findings compared to the global *Δe4−22* mice (Table 1)^45^. First, we demonstrate for the first time that deletion of *Shank3* in excitatory neurons of the cortex and hippocampus, and in selective MSN striatal population results in abnormalities across different behavioral domains. Contrary to the prediction, targeted *Shank3* deficiency in striatum by Dlx5/6-Cre, as well as, with the Drd1 or Drd2-specific Cre lines fails to produce the profound self-grooming phenotype observed in the global *Δe4−22* mice. In contrast, self-injurious skin lesions are obtained by targeting forebrain-specific *Shank3* deficiency with NEX-Cre. Second, we find that *Dlx5/6-Shank3* mice engage in perseverative and repetitive behaviors in the hole-board. Third, we observe that alterations in some behaviors (e.g., motor activity) are differentially affected among the various lines of *Shank3* conditional and global *Δe4−22* mice. These data suggest that *Shank3* may play disparate roles in specific cell types and these changes may regulate the neural circuits underlying ASD-like behaviors. Finally, *Shank3* deficiency in corticostriatal regions fails to reproduce the impaired social interaction, abnormal ultrasonic vocalizations, and deficient instrumental learning observed in the global *Shank3 Δe4−22* mice^45^. As a result, other brain regions appear to be more important in controlling these behaviors.

**Table 1:** Phenotypes of *Shank3* knockout mice^a^

Experiment	*Δe4−22*	*NEX-Shank3*	*Dlx5/6-Shank3*	*Drd1-Shank3*	*Drd2-Shank3*
Sociability	- 3 chamber	- 3 chamber	- 3 chamber	- 3 chamber	- 3 chamber
USVs	↓calls	- calls	- calls	- calls	- calls
	↓length	- length	- length	- length	- length
Repetitive behaviors	↑grooming	↑grooming	-grooming	- grooming	- grooming
	↑perseveration	-perseveration	↑perseveration	- perseveration	↑perseveration
Elevated zero maze	↑ exploration	- exploration	↑ exploration	↑ exploration	- exploration
Open Field	↓distance	↑distance	- distance	- distance	- distance
	↓rearing	↑ rearing	↓rearing	↓rearing	↑rearing
Instrumental Learning	↓lever pressing	-lever pressing	-lever pressing	N/A	N/A
Conditioned fear	↑context	- context	- context	N/A	N/A
	- cued	- cued	- cued		
Pre-pulse inhibition	↑ PPI	- PPI	↑ PPI	N/A	N/A
	↓startle	- startle	↓startle		
Rotarod	↓coordination	↓coordination	- coordination	- coordination	- coordination
Excitability	↑ spiking	N/A	N/A	↑ spiking	↑ spiking
Homer1b/c	↓ in striatum	- in any region	↓ in striatum	↓ in striatum	↓ in striatum
NMDAR currents	N/A	↑ hippocampus	N/A	N/A	N/A
GluN1/GluN2B	- in any region	↑ hippocampus	- in any region	N/A	N/A

^a^Targeting *Shank3* deletion by different Cre lines (NEX, Dlx5/6, Drd1, and Drd2) recapitulated different subsets of behavioral, electrophysiological, and biochemical features of the global *Δe4−22* mice. ↑ indicates an increase, ↓ indicates a decrease, - indicates that the mutant is similar to the wild-type control. N/A indicates not applicable, as the experiment was not conducted

However, our findings should also be interpreted with caution due to several caveats or confounding factors primarily related to the transcriptional complexity of *Shank3* as well as the specificity of Cre expression. The analyses of DNA in brain of conditional knockouts clearly demonstrated the occurrence of Cre-mediated recombination between *loxP* sites flanking exons 4–22 that are ~60 kb apart. We also detect a low percentage of recombination between exon 4–9 but not exons 10–22. The failure to detect exons 10–22 deletion indicates that the exon 4–22 deletion is reasonably sufficient in Cre-targeted cells. Quantitative analyses of *Shank3a-e* isoforms that are technically feasible were performed. The significant reduction of the *Shank3a* mRNA, the full length isoform in all lines of conditional knockout mice indicates the disruption of the major *Shank3* isoform. The variable or no significant reduction in the expression of other *Shank3* isoforms is not straight forward. The incomplete recombination between *loxP* sites and the persistent expression of *Shank3* isoforms in cells not targeted by the individual Cre line are likely to contribute to the expression of the residual isoforms. However, the analysis of *Shank3a-e* isoforms and quantification of immunoblots could not fully elucidate the exact nature and predicted complexity of isoform-specific disruption of *Shank3* mRNAs and protein isoforms because of a lack of the complete knowledge of *Shank3* mRNA composition and protein isoforms and the technical difficulties in examining cell type and isoform specific expression. Further study is warranted to elucidate the full spectrum of *Shank3* mRNA isoforms in different cell types and during development.

Excessive grooming in rodents is frequently used as an index for the stereotyped and compulsive behaviors in humans^69^. For instance, in a mouse model of obsessive-compulsive disorder (OCD), the *Sapap3* knockout mice can over-groom to an extent where lesions around the face and neck appear^70^. Interestingly, re-expression of *Sapap3* in the striatum of the homozygous mutants rescues the excessive grooming, suggesting that striatal dysfunction alone regulates this behavior. Further support for a striatal mechanism derives from experiments with Designer Receptors Exclusively Activated by Designer Drugs (DREADDs). Selective DREADD enhancement of activity in the D2-mediated indirect pathway, but not in the D1-mediated direct pathway, rescues the excessive grooming in isoform-specific *Shank3B* KO mice^71^. Other experiments, however, have indicated that this relationship may be more complex. An over-grooming phenotype can be evoked in EMX-Cre animals that are also co-expressed with the Cre inducible DIO-ChR2 vector through optogenetic activation of projections from the orbitofrontal cortex (OFC) to the striatum^26^. Additionally, optogenetic stimulation of OFC inputs to the striatum of *Sapap3* mice can suppress the excessive grooming, which indicates that cortical input may remediate the striatal deficit in these mutants^27^. Our present findings provide a support for cortical control by demonstrating that forebrain-specific loss of *Shank3* in *NEX-Shank3* leads to presentation of excessive self-grooming lesions. By comparison, the Dlx5/6-specific, Drd1-specific, or Drd2-specific *Shank3*-KO did not recapitulate the over-grooming phenotype in our study. Collectively, the existing studies and present findings suggest a more complex mechanism involving both cortical and striatal circuitry in excessive grooming of Shank3 models. Future work will examine cortical/striatal interactions in greater detail in the *Shank3* mice.

While targeting *Shank3* deletion to the striatum does not produce over-grooming, *Dlx5/6-Shank3* and *Drd2-Shank3* mutant mice engage in perseverative behaviors in the hole-board test. This dissociation between types of repetitive behavior such as stereotyped self-grooming and perseverative exploration has been reported for other ASD animal models^72^. Martos and colleagues^73^ selectively ablated striatal cholinergic interneurons and reported that social behavior was perturbed and that the repetitive exploratory behaviors were augmented while self-directed responses such as grooming were not affected. In the present study, the dissociation between excessive grooming and perseverative responses in *Dlx5/6-Shank3* and *Drd2-Shank3* mice indicate that different neural circuits underlie these behaviors and they emphasize the importance in carefully assessing behavioral endophenotypes in humans.

Some behavioral phenotypes in the global *Δe4−22* mice were not observed in the conditional animals. For instance, USVs and sociability were not perturbed in the conditional mice, suggesting that excitatory cortical neurons or inhibitory striatal neurons are not critical or sufficient to modulate these behaviors. This point, however, does not indicate that *Shank3* in these brain regions does not play some role in these responses because the USV study only examines one aspect of social communicative function and the sociability test does not evaluate the full range and complexity of social behavior in rodents. Other Shank3 mouse models also had relatively preserved sociability^74^. Aside from communicative function and social behavior being abnormal in the global *Δe4−22* mice^45^, they were impaired in instrumental learning—a form of operant conditioning hypothesized to involve corticostriatal circuits and is thought to involve reward learning^24,25^. We were surprised that forebrain or striatal specific *Shank3* deletion did not perturb instrumental learning. There are several possibilities why we obtained this result. *First*, brain regions other than or in addition to principle cells of the cortex or inhibitory neurons in the striatum may control this instrumental behavior. *Second*, given the diversity of cell types in brain, there may be a cellular sub-type that is not targeted by our Cre lines and is essential for expression of these behaviors as seen in global *Δe4−22* mice. Additionally, besides neurons, glia express also *Shank3* and they were not targeted for *Shank3* disruption in the four Cre lines used in our study^31^. *Third*, incomplete Cre-mediated recombination in the conditional *Shank3* knockouts may be sufficient to preserve their functioning. *Fourth*, since optogenetic cortical stimulation can override striatal dysfunction in *Sapap3* mice^27^, the loss of *Shank3* in either cortex or striatum may be compensated by the expression of this gene in reciprocal brain areas; thereby, preserving neural circuit function. *Finally*, all Cre mouse lines in this study have this gene expressed in mid-gestation. Since *Shank3* is expressed at an earlier time^31^, there may be some developmental role for *Shank3* that has yet to be identified. In this situation, *Shank3* expression prior to excision by Cre recombinase may account for certain behavioral phenotypes reported in the global *Shank3 Δe4−22* mice but absent in the conditional *Shank3* animals. Despite all of these caveats at a molecular level, our overall conclusions are supported by functional studies at the cellular level by whole cell recordings in striatum and cortex. Indeed, the cellular phenotypes in *Shank3* global knockout mice are well recapitulated in *Shank3* conditional mice and these indicate that the deficiency of *Shank3* in Cre targeted cells is sufficient.

The electrophysiological and biochemical characterizations of synaptic function and proteins in the conditional *Shank3* mice have replicated the key observations in the global *Shank3 Δe4−22* animals; however, they have revealed also some unexpected findings. The electrophysiological studies demonstrate that cell-type specific deletion of *Shank3* in Drd1-containing or Drd2-containing MSNs is sufficient to recapitulate the hyper-excitability phenotype reported in the cell-type indiscriminate recordings from the global *Δe4−22* mice^45^. Responses from neighboring cells of the opposite cell-type (i.e., D2 cells in Drd1 mutants or D1 cells in the Drd2 mutants) are indistinguishable from the WT controls, suggesting that the effects of *Shank3* deletion are cell autonomous despite likely reciprocal innervation^75^. This hyper-excitability state may arise from a compensatory mechanism in cells lacking *Shank3* which experience a reduction in synaptic transmission^45^. Indeed, global loss of some Shank3 isoforms can result in early hyper-excitability which may perturb the development of corticostriatal circuits^76^. Aside from the electrophysiological findings, our previous biochemical studies in the global *Δe4−22* mice found Homer1b/c protein to be significantly reduced in striatal PSDs^45^. This same result was recapitulated in the *Dlx5/6-Shank3*, *Drd1-Shank3* and *Drd2-Shank3* mutants, but not in the conditional mice where the cortical/hippocampal excitatory neurons were targeted. These findings indicate that Shank3 may form a scaffolding complex with Homer1b/c and metabotropic glutamate receptors in striatum with the exclusion of these functional interactions in other brain regions despite the abundance of these proteins in brain.

Hippocampal recordings from *NEX-Shank3* mice revealed an unexpected increase in NMDAR-mediated currents without alterations in AMPAR-currents. Intriguingly, this enhancement in both function and NMDAR proteins is contrary to what has been reported in various lines of global isoform-specific *Shank3* mice which show reduced NMDAR function and/or proteins^34,35,37,39,42,43^. Our electrophysiological results in the *NEX-Shank3* mice are consistent with the increase in NR1 and NR2B proteins in the hippocampus of these mutants; however, levels of these proteins are unchanged in the hippocampi of global *Shank3 Δe4−22* mice. This disparate pattern in protein levels and function of NMDAR-associated components between the *NEX-Shank3* and global *Shank3 Δe4−22* mice suggests that in the former model *Shank3* may modulate inhibitory inputs from some brain regions that affect hippocampal function. It should be emphasized that upregulation of NMDAR subunits has been observed also when Shank3 is selectively knocked down in developing hippocampal neurons^77^. Hence, these collective results indicate that selective alterations in Shank3 expression in different neuronal subtypes can exert biochemical and electrophysiological changes that may not be reflected in the global loss of this protein. In the future, more specific molecular and neural circuit studies may provide novel insights into their phenomenon.

In summary, our study of the first *Shank3* region-specific conditional mice have yielded several new insights into the function of this gene, as well as, illustrated the complexity of dissecting neural circuit mechanisms of behavior. From parallel analyses of multiple lines of conditional mice, we demonstrate that certain behavioral phenotypes in the global *Shank3 Δe4−22* mice can be attributed to brain-region or cell-type specificity. Intriguingly, selective targeting of *Shank3* in different brain regions or cells leads to distinct alterations in the interactions between Shank3 and other proteins in the PSD; these interactions appear to have functional consequences. However, interpretation of our findings may be complicated by a lack of complete knowledge of the complexity of *Shank3* mRNAs and protein isoforms in different cell types and during development. The presentation of abnormal behaviors was not always congruent between the global and the conditional *Shank3 Δe4−22* mice. Some of these distinctions may be attributed to the differences that Shank3 may play in the neural circuits subserving certain behaviors. Together, our results emphasize a need to examine in greater detail the separate and collective roles that different brain regions exert in the expression of Shank3-mediated behaviors. While our study has not identified the brain regions or specific cell types mediating all responses affected by *Shank3* deletion, it has provided new insights into the neural circuits responsible for ASD-associated stereotyped and repetitive behaviors and it has established a foundation for mechanistic studies to understand how loss of *Shank3* leads to synaptic and cellular dysfunctions associated with these behavioral phenotypes. Further study to elucidate the complete transcript structure and cell type specific expression of Shank3 isoforms in brain during the development is clearly warranted in future.

## Electronic supplementary material

Supplemental Figure 4

Supplemental Figure 3

Supplemental Figure 2

Supplemental Figure 1

Supplementary information

supplementary statistics dataset

## References

[1] American Psychiatric Association. (2013). Diagnostic and statistical manual of mental disorders.

[2] Autism Developmental Disabilities Monitoring Network. (2012). Prevalence of autism spectrum disorders--Autism and Developmental Disabilities Monitoring Network, 14 sites, United States, 2008. MMWR Surveill. Summ..

[3] DiCicco-Bloom E (2006). The developmental neurobiology of autism spectrum disorder. J. Neurosci..

[4] Langen M (2009). Changes in the developmental trajectories of striatum in autism. Biol. Psychiatry.

[5] Courchesne E, Pierce K (2005). Why the frontal cortex in autism might be talking only to itself: local over-connectivity but long-distance disconnection. Curr. Opin. Neurobiol..

[6] Courchesne E, Redcay E, Morgan JT, Kennedy DP (2005). Autism at the beginning: microstructural and growth abnormalities underlying the cognitive and behavioral phenotype of autism. Dev. Psychopathol..

[7] Uddin LQ, Supekar K, Menon V (2013). Reconceptualizing functional brain connectivity in autism from a developmental perspective. Front. Hum. Neurosci..

[8] Shepherd GM (2013). Corticostriatal connectivity and its role in disease. Nat. Rev. Neurosci..

[9] Fuccillo MV (2016). Striatal circuits as a common node for autism pathophysiology. Front Neurosci..

[10] Wood J, Ahmari SE (2015). A framework for understanding the emerging role of corticolimbic-ventral striatal networks in OCD-associated repetitive behaviors. Front. Syst. Neurosci..

[11] Dichter GS (2012). Reward circuitry function in autism spectrum disorders. Social. Cogn. Affect. Neurosci..

[12] Gunaydin LA, Kreitzer AC (2016). Cortico-basal ganglia circuit function in psychiatric disease. Annu. Rev. Physiol..

[13] Langen M, Kas MJ, Staal WG, van Engeland H, Durston S (2011). The neurobiology of repetitive behavior: of mice. Neurosci. Biobehav. Rev..

[14] Langen M, Durston S, Kas MJ, van Engeland H, Staal WG (2011). The neurobiology of repetitive behavior: and men. Neurosci. Biobehav. Rev..

[15] Sears LL (1999). An MRI study of the basal ganglia in autism. Progress. Neuropsychopharmacol. Biol. Psychiatry.

[16] Nickl-Jockschat T (2012). Brain structure anomalies in Autism spectrum disorder—a meta-analysis of VBM studies using anatomic likelihood estimation. Hum. Brain Mapp..

[17] Haznedar MM (2006). Volumetric analysis and three-dimensional glucose metabolic mapping of the striatum and thalamus in patients with autism spectrum disorders. Am. J. Psychiatry.

[18] Di Martino A (2011). Aberrant striatal functional connectivity in children with autism. Biol. Psychiatry.

[19] Delmonte S, Gallagher L, O’Hanlon E, McGrath J, Balsters JH (2013). Functional and structural connectivity of frontostriatal circuitry in Autism spectrum disorder. Front. Hum. Neurosci..

[20] Turner KC, Frost L, Linsenbardt D, McIlroy JR, Muller RA (2006). Atypically diffuse functional connectivity between caudate nuclei and cerebral cortex in autism. Behav. Brain Funct..

[21] Langen M (2014). Changes in the development of striatum are involved in repetitive behavior in autism. Biol. Psychiatry.

[22] Allsop SA, Vander Weele CM, Wichmann R, Tye KM (2014). Optogenetic insights on the relationship between anxiety-related behaviors and social deficits. Front. Behav. Neurosci..

[23] Calhoon GG, Tye KM (2015). Resolving the neural circuits of anxiety. Nat. Neurosci..

[24] Yin HH, Ostlund SB, Balleine BW (2008). Reward-guided learning beyond dopamine in the nucleus accumbens: the integrative functions of cortico-basal ganglia networks. Eur. J. Neurosci..

[25] Kelley AE (1999). Functional specificity of ventral striatal compartments in appetitive behaviors. Ann. N. Y. Acad. Sci..

[26] Ahmari SE (2013). Repeated cortico-striatal stimulation generates persistent OCD-like behavior. Science.

[27] Burguiere E, Monteiro P, Feng G, Graybiel AM (2013). Optogenetic stimulation of lateral orbitofronto-striatal pathway suppresses compulsive behaviors. Science.

[28] Jiang YH, Ehlers MD (2013). Modeling autism by SHANK gene mutations in mice. Neuron.

[29] Soorya L (2013). Prospective investigation of autism and genotype-phenotype correlations in 22q13 deletion syndrome and SHANK3 deficiency. Mol. Autism.

[30] Grabrucker AM, Schmeisser MJ, Schoen M, Boeckers TM (2011). Postsynaptic ProSAP/Shank scaffolds in the cross-hair of synaptopathies. Trends Cell Biol..

[31] Wang X, Xu Q, Bey AL, Lee Y, Jiang YH (2014). Transcriptional and functional complexity of Shank3 provides a molecular framework to understand the phenotypic heterogeneity of SHANK3 causing autism and Shank3 mutant mice. Mol. Autism.

[32] Leblond CS (2014). Meta-analysis of SHANK Mutations in Autism Spectrum Disorders: a gradient of severity in cognitive impairments. PLoS Genet..

[33] Bozdagi O (2010). Haploinsufficiency of the autism-associated Shank3 gene leads to deficits in synaptic function, social interaction, and social communication. Mol. Autism.

[34] Peca J (2011). Shank3 mutant mice display autistic-like behaviours and striatal dysfunction. Nature.

[35] Wang X (2011). Synaptic dysfunction and abnormal behaviors in mice lacking major isoforms of Shank3. Hum. Mol. Genet.

[36] Schmeisser MJ (2012). Autistic-like behaviours and hyperactivity in mice lacking ProSAP1/Shank2. Nature.

[37] Kouser M (2013). Loss of predominant Shank3 isoforms results in hippocampus-dependent impairments in behavior and synaptic transmission. J. Neurosci..

[38] Lee J (2015). Shank3-mutant mice lacking exon 9 show altered excitation/inhibition balance, enhanced rearing, and spatial memory deficit. Front. Cell. Neurosci..

[39] Duffney LJ (2015). Autism-like deficits in Shank3-deficient mice are rescued by targeting actin regulators. Cell Rep..

[40] Zhou Y (2016). Mice with Shank3 mutations associated with ASD and Schizophrenia display both shared and distinct defects. Neuron.

[41] Mei Y (2016). Adult restoration of Shank3 expression rescues selective autistic-like phenotypes. Nature.

[42] Speed HE (2015). Autism-associated insertion mutation (InsG) of Shank3 Exon 21 causes impaired synaptic transmission and behavioral deficits. J. Neurosci..

[43] Jaramillo TC (2015). Altered striatal synaptic function and abnormal behaviour in Shank3 Exon4-9 deletion mouse model of Autism. Autism Res..

[44] Durand CM (2007). Mutations in the gene encoding the synaptic scaffolding protein SHANK3 are associated with autism spectrum disorders. Nat. Genet..

[45] Wang X (2016). Altered mGluR5-Homer scaffolds and corticostriatal connectivity in a Shank3 complete knockout model of autism. Nat. Commun..

[46] Hulbert SW, Jiang YH (2017). Cellular and circuitry bases of Autism: lessons learned from the temporospatial manipulation of Autism genes in the brain. Neurosci. Bull..

[47] Bey AL, Jiang YH (2014). Overview of mouse models of autism spectrum disorders. Curr. Protoc. Pharmacol..

[48] Gong S (2007). Targeting Cre recombinase to specific neuron populations with bacterial artificial chromosome constructs. J. Neurosci..

[49] Monory K (2006). The endocannabinoid system controls key epileptogenic circuits in the hippocampus. Neuron.

[50] Goebbels S (2006). Genetic targeting of principal neurons in neocortex and hippocampus of NEX-Cre mice. Genesis.

[51] Rodriguiz RM, Colvin JS, Wetsel WC (2011). Neurophenotyping genetically modified mice for social behavior. Methods Mol. Biol..

[52] Rodriguiz, R. M. & Wetsel, W. C. Assessments of Cognitive Deficits in Mutant Mice. In: Levin, E. D., Buccafusco, J. J. (eds.). Animal Models of Cognitive Impairment. (CRC Press/Taylor & Francis, Boca Raton (FL), 2006) Chapter 12. Frontiers in Neuroscience. 21204369

[53] Guide for the Care and Use of Laboratory Animals, 8th edn, National Research Council (US) Committee for the Update of the Guide for the Care and Use of Laboratory Animals. (National Academies Press (US), Washington (DC), 2011). 21595115

[54] Zerucha T (2000). A highly conserved enhancer in the Dlx5/Dlx6 intergenic region is the site of cross-regulatory interactions between Dlx genes in the embryonic forebrain. J. Neurosci..

[55] Ohtsuka N (2008). Functional disturbances in the striatum by region-specific ablation of NMDA receptors. Proc. Natl Acad. Sci. USA.

[56] Shen HY (2008). A critical role of the adenosine A2A receptor in extrastriatal neurons in modulating psychomotor activity as revealed by opposite phenotypes of striatum and forebrain A2A receptor knock-outs. J. Neurosci..

[57] Yu J (2011). A sex-specific association of common variants of neuroligin genes (NLGN3 and NLGN4X) with autism spectrum disorders in a Chinese Han cohort. Behav. Brain Funct..

[58] Geng HY (2017). Erbb4 deletion from medium spiny neurons of the nucleus accumbens core induces schizophrenia-like behaviors via elevated GABAA receptor alpha1 subunit expression. J. Neurosci..

[59] Chao HT (2010). Dysfunction in GABA signalling mediates autism-like stereotypies and Rett syndrome phenotypes. Nature.

[60] Rozeske RR, Valerio S, Chaudun F, Herry C (2015). Prefrontal neuronal circuits of contextual fear conditioning. Genes Brain Behav..

[61] Madsen GF, Bilenberg N, Cantio C, Oranje B (2014). Increased prepulse inhibition and sensitization of the startle reflex in autistic children. Autism Res..

[62] Wurzman R, Forcelli PA, Griffey CJ, Kromer LF (2015). Repetitive grooming and sensorimotor abnormalities in an ephrin-A knockout model for Autism spectrum disorders. Behav. Brain Res..

[63] Swerdlow NR, Geyer MA, Braff DL (2001). Neural circuit regulation of prepulse inhibition of startle in the rat: current knowledge and future challenges. Psychopharmacology.

[64] Koch M (1999). The neurobiology of startle. Prog. Neurobiol..

[65] Graeff FG, Silveira MC, Nogueira RL, Audi EA, Oliveira RM (1993). Role of the amygdala and periaqueductal gray in anxiety and panic. Behav. Brain Res..

[66] Padilla-Coreano N (2016). Direct ventral hippocampal-prefrontal input is required for anxiety-related neural activity and behavior. Neuron.

[67] Yin HH (2016). The basal ganglia in action. Neuroscientist.

[68] Ade KK, Wan Y, Chen M, Gloss B, Calakos N (2011). An improved BAC transgenic fluorescent reporter line for sensitive and specific identification of striatonigral medium spiny neurons. Front. Syst. Neurosci..

[69] Kalueff AV (2016). Neurobiology of rodent self-grooming and its value for translational neuroscience. Nat. Rev. Neurosci..

[70] Welch JM (2007). Cortico-striatal synaptic defects and OCD-like behaviours in Sapap3-mutant mice. Nature.

[71] Wang W (2017). Striatopallidal dysfunction underlies repetitive behavior in Shank3-deficient model of autism. J. Clin. Invest..

[72] Moy SS (2014). Repetitive behavior profile and supersensitivity to amphetamine in the C58/J mouse model of autism. Behav. Brain Res..

[73] Martos YV, Braz BY, Beccaria JP, Murer MG, Belforte JE (2017). Compulsive social behavior emerges after selective ablation of striatal cholinergic interneurons. J. Neurosci..

[74] Yang M (2012). Reduced excitatory neurotransmission and mild autism-relevant phenotypes in adolescent Shank3 null mutant mice. J. Neurosci..

[75] Taverna S, Ilijic E, Surmeier DJ (2008). Recurrent collateral connections of striatal medium spiny neurons are disrupted in models of Parkinson’s disease. J. Neurosci..

[76] Peixoto RT, Wang W, Croney DM, Kozorovitskiy Y, Sabatini BL (2016). Early hyperactivity and precocious maturation of corticostriatal circuits in Shank3B mice. Nat. Neurosci..

[77] Halbedl S, Schoen M, Feiler MS, Boeckers TM, Schmeisser MJ (2016). Shank3 is localized in axons and presynaptic specializations of developing hippocampal neurons and involved in the modulation of NMDA receptor levels at axon terminals. J. Neurochem..

