# Supplementary material for: Brain region-specific disruption of Shank3 in mice reveals a dissociation for cortical and striatal circuits in autism-related behaviors
Source: Transl Psychiatry. 2018 Apr 27;8:94. doi: 10.1038/s41398-018-0142-6 (PMC5919902; doi:10.1038/s41398-018-0142-6)
Supplement: Supplementary file 5 — Supplementary information [file 41398_2018_142_MOESM5_ESM.docx]

**Supplemental Information**

# Detailed Methods & Materials

## Genotyping and PCR confirmation of loss of *Shank3*

Primers CRE-F (5'-caacgagtgatgaggttcgcaag-3') and CRE-R (5'-atatttacattggtccagccaccagc-3') were designed to amplify a 907 bp product found within the conserved Cre sequence and were used to determine the presence of Cre with a 30 s denaturing step at 94ºC, 30 s annealing step at 60ºC, and a 45 s extension step at 72ºC for 35 cycles. Primers FLP-NEO (5'-gggaggattgggaagacaat-3'), BFLP-F (5'-atctaccaccccctggtctc-3'), and SH-BFLP-R (5'- ccgaggtaatcaagacc-3') were used to distinguish the wild-type (WT; 315 bp) from the floxed (240 bp) *Shank3* allele with a 30 s denaturing step at 94ºC, 30 s annealing step at 58ºC, and a 30 s extension step at 72ºC for 35 cycles. To identify recombination of the first and third loxP sites (Δe4-22), primers SHXY-F (5'-ttgcatctgggacctactcc-3'), PURO-F (5'-gcaacctccccttctacgag-3'), and SH3-3’-R (5'-aaagcactgactcctctcttgg-3') were used to amplify a 673 bp product when the floxed allele was intact and a 599 bp product when recombination had occurred with a 30 s denaturing step at 94ºC, 30 s annealing step at 58ºC, and a 45 s extension step at 72ºC for 35 cycles.

## Genomic DNA quantitative-PCR

Genomic DNA was prepared using proteinase K lysis and ethanol precipitation from dissected brains of WT and conditional *Shank3* KO littermates, as well as standard samples from global *Shank3 ∆e4-22*^+/+^, *∆e4-22*^+/-^ , and *∆e4-22*^-/-^  brains. Primers QPCR-F (5'- aggacaggtgacagtcaatgg-3') and QPCR-R (5'-gcctagcatacctgccttca-3') were used to amplify a 270 bp fragment when *Shank3* exons 4-22 had been recombined using real-time quantitative PCR with iQ SYBR Green Supermix (Bio Rad, Hercules, CA) on a LightCycler 480 instrument (Roche Diagnostics, Mannheim, Germany) per manufacturer’s instructions. All qPCR results were normalized to genomic DNA primers for *Oxtr*, and relative recombination was calculated using a formula derived from linear fitting of a standard curve from global *Shank3* *∆e4-22* standards.

## RNA isolation and Reverse transcriptase quantitative-PCR

RNA was prepared from dissected cortical, hippocampal, and striatal samples using the SV Total RNA Isolation system (Promega, Madison, WI). Reverse transcription was performed using the SuperScript^TM^ III first-strand synthesis system (Thermo Fisher, Waltham, MA). Previously described primers^1^ amplifying *Shank3a, Shank3b, Shank3c, Shank3d,* and *Shank3e* mRNA isoforms were used with iQ SYBR Green Supermix on a LightCycler 480 instrument per manufacturer’s instruction.

## Animal husbandry

Mice were housed 4-5 mice per cage (unless otherwise specified) on a 14:10 hr light/dark cycle with food and water provided *ad libitum*. Most of the behavioral testing occurred during the light cycle. LabSan 256CPQ solution (Sanitation Strategies LLC, Williamston, MI) was used for sanitizing the behavioral equipment between mice. Animal husbandry and behavioral testing were conducted with approved protocols from the Duke University Animal Care and Use Committee, which were in accordance with the NIH Guidelines for the Care and Use of Laboratory Animals ^2^. Mice developing lesions were euthanized according to IACUC policy and were excluded from subsequent tests.

## Sociability test

Mice were examined for sociability as described ^3^. Briefly, the testing was conducted in 2 phases. Test phase 1 began when a subject mouse (i.e., WT or KO) was placed into the center of the chamber and given free-access to the entire apparatus. In this phase, the test chamber was equipped with identical non-social stimuli placed into wire-mesh cages, each located in the opposite outer thirds of the chamber. After 10 min, the subject mouse was removed and an adult female C3H/HeJ mouse (Stock No. 000659; Jackson Laboratories) was introduced into one of the wire-mesh cages. Test phase 2 (social affiliation) began with reintroduction of the subject mouse into chamber and testing continued over 10 min. All tests were filmed and the digital videos were analyzed subsequently using EthoVision XT software version 9 (Noldus Information Technology, Leesburg, VA) that included the duration of contacts with each stimulus cage. Preference scores were calculated, where time spent with one stimulus (non-social stimulus 1 or the social stimulus) was subtracted from the time spent with the other stimulus (non-social stimulus 2 or the non-social stimulus, respectively) and divided by the total time spent exploring both stimuli. Positive scores indicated a preference for the social relative to the non-social stimulus, whereas negative scores reflected a preference for the non-social stimulus; and scores approximating “0” indicated no preference. See Supplemental Table 2.

## Resident-intruder test

*Shank3* mice were housed individually for >14 days before testing ^4^. All testing was performed under red-light illumination (<5 lux) 2-6 hr after the onset of the dark cycle. Subject *Shank3* mice were paired with non-familiar partner C3H/HeJ mice (Jackson Laboratories) of the same age, sex, and approximate weight. The partner C3H/HeJ mouse was introduced into the home-cage of the *Shank3* subject mouse and they were permitted to interact for 5 min. All tests were filmed and the videos were scored by blinded trained observers using a simplified ethogram based on our previous work ^3, 4^ which demonstrated that time spent in non-reciprocated social approach was the only variable significantly different for *Shank3 e4-22* mice out of a great number of more detailed behavioral observations. Here, social behaviors were collapsed into two categories: 1) total time spent in bidirectional social interaction, which consists of one mouse engaging the other (irrespective of initiator and type of social behavior) and the other animal reciprocating the social behavior, and 2) total time spent in non-reciprocated subject-to-partner interaction, which comprises conditions when the *Shank3* mouse engaged the C3H partner, but the partner did not reciprocate the behavior.

## Adult ultrasonic vocalizations (USVs)

Ultrasonic communication was examined as described ^3^. Briefly, following a week of reproductive experience, male mice were habituated to recording chambers for 5 min then exposed to a novel estrous female for 5 min. USVs were recorded as waveform audio files and analyzed subsequently using Avisoft Bioacoustics SASLab Pro software (Glienicke, Germany). Mice that did not call were excluded from the subsequent analyses of call duration, peak amplitude, and peak frequency.

## Self-grooming

Individual animals were acclimated to clean home cages for 5 min prior to filming (MediaRecorder2; Noldus Information Technologies). Mice were filmed for 10 min and natural grooming behavior was hand-scored using Observer 9 XT (Noldus Information Technologies).

## Skin lesions

Individual animals in the behavioral cohorts were observed at minimum bi-weekly for emergence of skin lesions from transfer to the behavioral facility post-weaning until completion of the behavioral battery and sacrifice. Skin lesions were defined as loss of hair which progressed to redness and irritation of the underlying skin and were either present or absent for each animal. Lesions were observed exclusively to the back of the head and exclusively in *NEX-Shank3* mice.

## Hole-board test

Animals were examined in the hole-board test for 5 min as described ^3^. Briefly, mice were allowed 5 min exploration of a 16-hole-board apparatus. Animals were filmed with a digital video camera and hand-scored for the numbers of nose-pokes and the location of each nose-poke. Back-to-back nose pokes were defined as when the animal made two or more consecutive visits to the same hole.

## Fear conditioning

Animals were tested for contextual and cued fear conditioning as described ^3, 5^. Briefly, day 1 consisted of conditioning using the single presentation of a 30 s tone (conditioned stimulus or CS) terminating simultaneously with a 2 s foot-shock (unconditioned stimulus or UCS). Day 2 examined contextual fear by returning the mice returning to the chamber in which they had been conditioned for 5 min in the absence of the CS and UCS. Day 3 examined cued fear, with animals being placed into novel chambers in the absence of the CS for 2 min followed by the presence of the CS for the final 3 min. For all tests, behavior was videotaped and scored for freezing in an automated fashion by FreezeScan software (Cleversys, Reston VA).

## Instrumental learning

A continuous reinforcement paradigm to assess instrumental learning was conducted as described ^3^. Briefly, following food restriction to achieve 85% free-feeding weights, mice were given daily sessions in operant chambers (MedAssociates, St. Albans, VT) where each lever press earned a 5 s presentation of a sweetened, condensed milk reward. Sessions terminated following 100 lever presses or 60 min, whichever occurred first.

## Prepulse inhibition (PPI)

PPI was examined as described using the SRL-Lab startle response system and software (San Diego Instruments, San Diego, CA) ^3^. The prepulse trials included 20 ms prepulse stimuli that were 4, 8, or 12 dB above the white-noise background (64 dB) and this was followed 100 ms later with a 40 ms 120 dB white-noise pulse stimulus. Each test consisted of 74 trials with 30 pulse-alone trials, 8 null trials, and 36 prepulse-pulse trials. Responses were measured as the maximum startle response (mAmp platform displacement) following presentation of the pulse or startle stimulus, or during the null trials composed of the white-noise background. PPI was calculated as the ratio of the startle responses on prepulse trials to startle-only trials, subtracted from 1 and expressed as a percentage: [1 - (prepulse-pulse trials/pulse-alone trials)*100].

## Elevated zero maze

Anxiety-like behaviors were assessed in the elevated zero maze (see Pogorelov et al., 2005; Welch et al., 2007). Mice were introduced into the closed area of the maze and given 5 min of free exploration under dim (40-60 lux) illumination. Performance was scored by Ethovision XT 7 (Noldus Information Technologies) using a high-resolution camera suspended 180 cm above the center of the maze. Tracking profiles were generated by Ethovision XT software and were used to measure the latency to enter the open areas as well as the numbers of closed-to-open-to-closed area transitions.

## Open field activity

Spontaneous activity in the open field was conducted over 1 hr in an automated Omnitech Digiscan apparatus (AccuScan Instruments, Columbus, OH). Accuscan software scored the distance traveled, vertical activity (beam-breaks), and time spent in the center zone.

## Accelerating rotarod performance

Balance and coordination were examined using a rotarod (Med-Associates) as described ^3^. The rotarod accelerated from 4 to 40 rpm over 5 min and mice were given 4 successive 5-min trials with an inter-trial interval of 30 min. Trials were terminated when the mouse fell from the rod or at 300 s.

## Whole-cell patch clamp recording from brain slices

Adult mice (5-10 months) were deeply anesthetized with halothane (Sigma, St. Louis, MO) and decapitated. The brains were quickly removed from the skulls and placed in ice-cold (<4°C) modified artificial cerebrospinal fluid (ACSF) containing (in mM) 120 NaCl, 3.3 KCl, 1.23 NaH_2_PO_4_, 1 MgSO_4_, 2 CaCl_2_, 25 NaHCO_3_, and 10 d-glucose at pH 7.3, previously saturated with 95%O_2_-5%CO_2_. Coronal striatal slices or sagittal hippocampal slices were sectioned at 300 μm using a Vibratome series 1000 sectioning system (Vibratome, St. Louis, MO). The brain slices were allowed to equilibrate for ≥1 h at 33°C in the ACSF solution that was continuously bubbled with a mixture of 95% O_2_-5% CO_2_ gas. Slices were kept in bubbled ACSF at room temperature (22–24°C) until the recordings were initiated. The slices were perfused by bubbled ACSF at 29-30°C with a 2 ml/min flow rate. The slice was visualized with infrared differential interference contrast (DIC; Zeiss Axio Examiner D1), using an upright microscope, with a ×40 water-immersion objective, and displayed on a monitor. Recordings of action potentials were performed from medium spiny neurons (MSNs) in the dorsolateral striatum. After identifying direct pathway MSNs (D1) by the tdTomato signal, tdTomato-negative neurons were assumed to be indirect pathway MSNs (D2). Synaptic currents were recorded from hippocampal CA1 pyramidal neurons. Recordings were made by whole cell patch recording using the Multiclamp 700B amplifier (Molecular Devices, Axon Instruments Inc., Union City, CA). The signals were filtered at 10 kHz and acquired using Digidata 1440A and pClamp 10.7 (Axon Instruments, Molecular Devices). Patch pipettes were borosilicate glass capillaries (1.5 mm OD, 1.1 mm ID, Sutter Instrument, Novato, CA), pulled on a Flaming/Brown Micropipette Puller (Sutter Instrument, Model P-87) to produce electrodes with 3–4 MΩ resistance. The pipette solution for current-clamp experiments consisted of (in mM) 130 K-gluconate, 5 KCl, 2 MgCl_2_, 0.2 EGTA, 10 HEPES, 4 Mg-ATP, 0.5 Tris-GTP, and 10 phosphocreatine, pH adjusted to 7.3 with KOH (290 mosM). For voltage-clamp experiments (evoked post synaptic currents, eEPSCs), the patch pipettes were filled with (in mM) 130 CsMeSO4, 1 MgCl_2_, 10 HEPES, 0.5 EGTA, 4 Mg-ATP, 0.5 Na-GTP, 10 phosphocreatine, and 4 lidocaine N-ethyl bromide (QX314), pH adjusted to 7.3 with CsOH (290 mosM). The access resistance was monitored throughout each experiment, and only recordings with stable access and holding currents for at least 3 min were used. Data were excluded from the analysis when the series resistance changed by >15%. The liquid junction potential was estimated to be 15.9 mV for the normal ACSF solution and was not corrected. Input resistance was calculated from membrane voltage deflection, evoked by 600-ms hyperpolarizing current injections (0 to -300 pA in steps of 50 pA) and calculated from the measured slope. Single action potentials were elicited by 10 ms depolarizing current injections with 10-pA increments in current-clamp mode.  Evoked action potentials were elicited by injecting a depolarizing current from 0 pA to 400 pA for 600 ms duration in 10-pA increments. Synaptic currents were evoked by stimuli at 0.1 Hz in 0.25 ms duration through a stimulating electrode placed within 60-100 µm from the neuron under voltage-clamp recording. The eEPSCs were conducted in the presence of the GABA_A_ receptor antagonist picrotoxin (20 µM). For input-output response, EPSCs were elicited by a series of pulses with different stimulation intensities (50-300 µA) delivered at 0.1 Hz. To obtain a NMDAR- to AMPAR-EPSC ratio, AMPAR-EPSCs were first recorded in the ACSF solution (containing picrotoxin) at a -70 mV holding potential. Then DNQX (20 µM) was added and NMDAR-EPSCs were recorded with the same stimulation pulse at a holding potential of +40 mV.

## Antibodies

The GluN2B (N59/36) antibodies were purchased from UC Davis/NIH NeuroMab. The actin (sc-1615), Homer1b/c (sc-20807), C-terminal Shank3 (sc-30193), and all HRP-conjugated secondary antibodies were obtained from Santa Cruz Biotechnology (Santa Cruz, CA). The GluN2A (07-632) antibody was from Millipore (Billerica, MA), while the GluA1 (ab31232) and β-tubulin III (ab18207) antibodies were purchased from Abcam (Cambridge, MA). The Bassoon (SAP7F407) antibody was from Stressgen (Ann Arbor, MI).

## Preparation of crude PSD proteins

Isolation of crude PSDs have been described previously ^3^. Briefly, tissues from different brain regions were homogenized in a HEPES-buffered sucrose solution (0.32 M sucrose, 4 mM HEPES, pH 7.4) and centrifuged at 800 × g for 10 min at 4°C. The cloudy supernatants were transferred to a new set of tubes and subjected to 12,000 × g centrifugation for 15 min to yield the pellet (P2) which was lysed in water, buffered with HEPES (pH 7.4) to 4 mM, and submitted to centrifugation at 20,500 × g for 30 min to yield the P3 synaptosomal membrane (SPM) fraction. The SPM was re-suspended in a buffer containing 50 mM HEPES (pH 7.4), 2 mM EDTA, and 0.5% Triton X-100. After 15 min of mixing by rotation at 4°C, the crude PSD-I fraction was obtained by centrifugation at 20,500 × g for 20 min at 4°C. The crude PSD pellet was dissolved in 1% SDS-PBS for further quantitative immunoblot analysis.

## Quantitative immunoblot analysis

Equal amounts of proteins from whole cell lysates or crude PSD fractions were separated by SDS-PAGE. Proteins were transferred to PVDF membranes (Bio-Rad, Hercules, CA). After blocking the membrane at room temperature for 1 hr in 0.02M Tris-buffered saline (TBS; pH 7.4) with 5% non-fat milk, the blots were incubated with the corresponding primary antibodies at 4°C overnight (see Supplemental Table 3 for concentrations). The blots were washed in TBS containing 0.1% Tween-20 (TBST) and incubated with HRP-conjugated secondary antibodies (Santa Cruz Biotechnology) for 60 min at room temperature. Following 3 washes in TBST, the blots were incubated with ECL reagent (GE Healthcare Life Sciences, Piscataway, NJ) and exposed to Kodak X-ray film (Rochester, NY). For quantification, films were scanned by a UVP image system (Upland, CA), the gray values of proteins were analyzed by ImageJ software (NIH, Bethesda, MD), and normalized to that of the corresponding internal loading controls (i.e., actin or β-tubulin III).

## Statistical analyses

When comparisons between genotypes were made for within-subject measurements across different phases of the same test (e.g., test days, locations within a test arena, or different intensities of stimuli), the data were analyzed with repeated measures ANOVA (RMANOVA). For post-hoc comparisons, t-tests with a Bonferroni-correction for multiple comparisons were applied. Statistical significance was defined as *p*<0.05.

# Supplemental Table 1. The cohorts used and the order of behavioral testing of *Shank3* conditional knockout mice

| **Cohort 1: Drd1 & Drd2** | **Cohort 2: Drd1 & Drd2** | **Cohort 3: NEX & Dlx5/6** | **Cohort 4: NEX & Dlx5/6** | **Cohort 5: NEX & Dlx5/6** | **Cohort 6: Drd1** | **Cohort 7: Shank3 Δe4-22^2^** |
| --- | --- | --- | --- | --- | --- | --- |
| n=9 per genotype (4 groups) | n=5-9 per genotype (4 groups) | n= 9-12 per genotype (4 groups) | n= 10-12 per genotype (4 groups) | n=4-5 per genotype (4 groups) | n=5 per genotype (2 groups) | n=9-10 per genotype (2 groups) |
| Elevated Zero Maze | Elevated Zero Maze | Elevated Zero Maze | Instrumental Learning | Open Field Test | Elevated Zero Maze | Elevated Zero Maze |
| Open Field Test | Open Field Test | Open Field Test | Ultrasonic Vocalizations^1^ | Self-grooming | Skin lesion observation (throughout) | Open Field Test |
| Holeboard | Holeboard | Holeboard | Skin lesion observation (throughout) | Holeboard (Dlx5/6 only) |  | Self-grooming |
| Self-grooming | Self-grooming | Self-grooming |  | Skin lesion observation (throughout) |  | Rotarod |
| Rotarod | Rotarod | Rotarod |  |  |  |  |
| Sociability | Sociability | Sociability |  |  |  |  |
| Ultrasonic Vocalizations^1^ | Ultrasonic Vocalizations^1^ | Pre-Pulse Inhibition |  |  |  |  |
| Skin lesion observation (throughout) | Skin lesion observation (throughout) | Ultrasonic Vocalizations^1^ |  |  |  |  |
|  |  | Resident Intruder |  |  |  |  |
|  |  | Fear Conditioning |  |  |  |  |
|  |  | Skin lesion observation (throughout) |  |  |  |  |

^1^Ultrasonic vocalizations were tested in male mice only.

^2^Sociability, Pre-Pulse Inhibition, Ultrasonic vocalizations, and Fear Conditioning were previously performed and reported^3^. Data presented in this manuscript from *Shank3 Δe4-22* mice has been reanalyzed for comparison here to the conditional knockout lines.

# Supplemental Table 2. Results of additional behavioral testing in *Shank3* conditional knockout mice

| Test | *NEX-Shank3* | *Dlx5/6-Shank3* | *Drd1-Shank3* | *Drd2-Shank3* |
| --- | --- | --- | --- | --- |
| **Sociability** |  |  |  |  |
| NS-NS^1^ preference | +/+ -0.04 ± 0.04  -/- 0.06 ± 0.08 | +/+ 0.06 ± 0.08  -/- 0.07 ± 0.07 | +/+ -0.03 ± 0.05  -/- -0.05 ± 0.05 | +/+ 0.01 ± 0.03  -/- -0.02 ± 0.05 |
| Duration (s) with stimuli in NS-NS | +/+ 217.1 ± 7.2  -/- 191.0 ± 9.2 | +/+ 179.6 ± 7.8  -/- 191.0 ± 5.0 | +/+ 197.4 ± 15.0  -/- 198.9 ± 12.4 | +/+ 218.1 ± 11.3  -/- 236.1 ± 14.7 |
| Duration (s) with stimuli in NS-S1^2^ | +/+ 285.3 ± 16.9  -/- 271.9 ± 15.5 | +/+ 258.7 ± 15.0  -/- 262.2 ± 13.8 | +/+ 277.0 ± 15.9  -/- 255.0 ± 15.6 | +/+ 284.1 ± 10.9  -/- 292.2 ± 14.1 |
|  |  |  |  |  |
| **Resident-Intruder** |  |  |  |  |
| Bi-directional interaction (s) | +/+ 41.7 ± 12.4  -/- 24.5 ± 4.0 | +/+ 12.1 ± 1.3  -/- 28.4 ± 6.7 * |  |  |
| Non-reciprocated interaction (s) | +/+ 154.3 ± 18.3  -/- 144.8 ± 20.6 | +/+ 109.0 ± 24.2  -/- 135.7 ± 26.1 | N/A | N/A |

^1^NS = non-social

^2^S1 = social stimulus, i.e. a novel mouse, presented in the 2^nd^ phase of the sociability assay

^*^ Two-tailed t-test, t(13)=-2.236, p=0.044, n=7 WT, 8KO

**No other significant genotype differences were found within these tests across any of the strains.**

|  |  |
| --- | --- |
|  |  |
|  |  |
|  |  |
|  |  |

# Supplemental Table 3. The concentrations of antibodies used for quantitative immunoblotting

| **Antibody Name** | **Concentration** | **Manufacturer** |
| --- | --- | --- |
| actin (sc-1615) | 1:5,000 | Santa Cruz Biotechnology |
| Homer1b/c (sc-20807) | 1:10,000 | Santa Cruz Biotechnology |
| C-terminal Shank3 (sc-30193) | 1:5,000 | Santa Cruz Biotechnology |
| GluN2B (N59/36) | 1:3000 | UC Davis/NIH NeuroMab |
| GluN2A (07-632) | 1:1000 | Millipore |
| GluA1 (ab31232) | 1:1000 | Abcam |
| β-tubulin III (ab18207) | 1:20,000 | Abcam |
| anti-goat HRP | 1:5,000 | Santa Cruz Biotechnology |
| anti-rabbit HRP | 1:5,000 | Santa Cruz Biotechnology |
| anti-mouse HRP | 1:5,000 | Santa Cruz Biotechnology |

# Supplemental Table 4. Intrinsic membrane properties and single action potential (AP) characteristics from striatum D1 and D2 neurons

|  | D1 WT | | | D1 KO | | D2 WT | | D2 KO | |
| --- | --- | --- | --- | --- | --- | --- | --- | --- | --- |
|  | D1 | D2 | | D1 | D2 | D1 | D2 | D1 | D2 |
| Input resistance  (MΩ) | 48.9 ±3.4  (11) | 69.1± 4.5  (13) | 61.5±3.2*  (16) | | 60.5 ±7.9  (6) | 54.2±4.9  (12) | 63.7±2.9  (14) | 49.4±2.3  (17) | 67.6±2.3  (16) |
| RM potential (mV) | -74.7 ± 1.0 | -72.2 ±1.1 | -69.1 ±1.3* | | -70.0±2.1 | -76.9 ±0.9 | -71.3 ±1.3 | -75.6±0.9 | -71.5 ±0.6 |
| Current threshold (pA) | 432.3 ±28.1 | 308.3 ±46.1 | 286.4±32.1* | | 320±45.7 | 427.5 ±24.8 | 300.0 ±35.4 | 465.0 ±22.6 | 313.1±27.2 |
| AP threshold (mV) | -47.8 ±1.3 | -49.0 ±1.5 | -46.4 ±1.5 | | -47.4 ±2.1 | -50.7 ± 0.9 | -47.9 ±0.9 | -47.4 ±0.5 | -49.4 ±1.4 |
| AP amplitude (mV) | 119.3 ±1.3 | 114.5 ±2.4 | 116.6 ±2.7 | | 118.3±3.2 | 120.3 ± 0.7 | 112.7±1.5 | 115.8 ±1.0 | 111.0 ±1.2 |
| AP duration (ms) | 0.97 ±0.02 | 0.95 ±0.04 | 0.98 ±0.03 | | 0.98 ±0.06 | 0.83 ± 0.01 | 1.0 ± 0.04 | 0.94 ±0.05 | 0.96 ±0.02 |
| AP rise time (ms) | 0.24 ±0.01 | 0.23 ±0.01 | 0.26 ±0.01 | | 0.24 ±0.01 | 0.20 ±0.01 | 0.24 ±0.01 | 0.27 ±0.01 | 0.26 ±0.01 |
| AP decay time (ms) | 0.72 ±0.03 | 0.72 ±0.04 | 0.77 ±0.02 | | 0.81 ±0.06 | 0.68 ±0.02 | 0.80 ± 0.03 | 0.70 ±0.05 | 0.69 ±0.02 |
| N | 13 | 12 | 14 | | 8 | 12 | 12 | 12 | 16 |

*p<0.01

# Supplemental Figure Legends

**Supplemental Figure 1: Quantitative PCR for Cre-mediated recombination between loxP sites of *Shank3 e4-22^flox^***

**(a)** PCR-based detection of recombination of first and third loxP sites (Δe4-22) detected in the cortex (CX) and hippocampus (HP), but not striatum (ST) (2-way ANOVA, main effects of genotype and region, *p-values* <0.001, genotype x region interaction, *p-value* <0.001), of NEX-Cre *Shank3* floxed mice (*NEX-Shank3*); n=3/genotype/region. **(b)** PCR-based detection of recombination of Δe4-22 detected in ST, but not CX or HP (2-way ANOVA, main effects of genotype and region, *p-values* <0.001, genotype x region interaction, *p*<0.001) of Dlx5/6-Cre *Shank3* floxed mice (*Dlx5/6-Shank3*); n=2-4/genotype/region. **(c)** PCR-based detection of recombination of Δe4-22 detected in ST, CX and HP (2-way ANOVA, main effects of genotype and region, *p-values* <0.001, genotype x region interaction, *p*<0.001), of Drd1-Cre  *Shank3* floxed mice (*Drd1-Shank3*); n=3/genotype/region. **(d)** PCR-based detection of recombination of Δe4-22 detected in ST, but not CX or HP (2-way ANOVA, main effects of genotype and region, *p-values* <0.001, genotype x region interaction, *p*<0.001) of Drd2-Cre *Shank3* floxed mice (*Drd2-Shank3*); n=3/genotype/region. **(e-h)** Quantitative PCR for different Shank3 isoforms in different brain regions of NEX-(e), Dlx5/6-(f), Drd1-(g), and Drd2-Shank3(h) conditional knockout mice respectively. n=3/genotype/region. Data were analyzed using 2-way ANOVA, see details in supplemental statistical sheet. For all panels, **p*<0.05, compared to wild-type controls in Bonferroni-corrected post-hoc comparisons. All data are expressed as means ± SEM.

**Supplemental Figure 2: Region-specific deletion of Shank3 does not recapitulate many of the learning and locomotor comorbidities of global *Δe4-22*** **mice**

**(a-b)** Conditioned Fear. (a) During contextual recall, global *Δe4-22* mice showed a small, but significant increase in freezing during contextual recall (t-test, *p=*0.031). Both *NEX-Shank3* and *Dlx5/6-Shank3* mice showed similar rates of freezing to their wild-type (+/+) littermates; n=7-12/genotype. (b) During cued recall during presentation of the cued stimulus, all genotypes showed significantly increased freezing relative to the pre-tone period (RMANOVA, main effect of phase p<0.001), and no genotype differences were found within each strain; t-tests, n=7-12/genotype. **(c-d)** Instrumental learning. Unlike what is seen in the global *Δe4-22* mice, *NEX-Shank3* (c) and *Dlx5/6-Shank3* mice (d) demonstrated comparable, but variable, performance in an instrumental learning task as their wild-type littermates (RMANOVA, main effect of trial *p*<0.001, no significant effect of genotype or trial x genotype interaction); n=10-13/genotype. **(e)** Anxiety-like behavior in the open field. Global *Δe4-22* mice spent less time in the center of the open field (t-test, *p*=0.050), whereas no significant differences were seen in *NEX-Shank3, Dlx5/6-Shank3, Drd1-Shank3*, or *Drd2-Shank3* mice; n=12-18/genotype. **(f-i)** Only *NEX-Shank3* mice differed in their performance (RMANOVA, trial x genotype interaction *p*=0.01), which was worse on the second trial (Bonferroni corrected comparison *p*<0.05); n=10-12/genotype. (g-i) No genotype differences were detected within the other three strains of conditional knockouts; n=9-18/genotype. For all panels, **p*<0.05, compared to wild-type controls. All data are expressed as means ± SEM.

**Supplemental Figure 3: Loss of *Shank3* in direct and indirect medium spiny neurons does not alter the membrane excitability of non-targeted neighboring cells**

**(a)** Example micrograph of patched medium spiny neuron in the dorsolateral striatum. Putative D1 (Drd1-tdTomato positive) and D2 (tdTomato negative) neurons were identified and recorded from the same slice. Example traces of overlay of single action potentials evoked with a 10 ms current injection in D1 MSNs neurons from *Drd1-Shank3* WT (+/+) and KO (-/-) mice. **(b)** Summarized data for current threshold to induce action potentials in D1 and D2 MSNs neurons from *Drd1-Shank3* WT (+/+) and KO (-/-) mice (t-test; *p=0.003). **(c)** Summarized data for current threshold to induce action potentials in D1 and D2 MSNs neurons from *Drd2-Shank3* WT (+/+) and KO (-/-) mice. **(d)** Summarized data for the numbers of evoked action potentials at the indicated amplitudes of current injection from D2 MSNs from *Drd1-Shank3* WT (+/+) and KO (-/-) mice.  **(e)** Summarized data for the numbers of evoked action potentials at the indicated amplitudes of current injection from D1 MSNs from *Drd2-Shank3* WT (+/+) and KO (-/-) mice. All data are expressed as means ± SEM.

**Supplemental Figure 4: Loss of *Shank3* perturbs NMDAR function in hippocampus.**

**(a-d)** Recordings from hippocampal CA1 neurons from *NEX-Shank3* mice. **(a)** Example traces of evoked NMDAR EPSCs recorded at +40 mV in hippocampal CA1 neurons form *NEX-Shank3* WT (+/+) and KO (-/-) mice. **(b)** Input-output curves of NMDAR-EPSCs in response to a series of stimulation intensities in CA1 hippocampal neurons from *NEX-Shank3* WT (+/+) and KO (-/-) mice (2-way ANOVA, main effects of genotype and stimulation, *p-values* <0.02, genotype x stimulation interaction not significant). **(c)** Example traces of evoked AMPAR EPSCs recorded at -70 mV in hippocampal CA1 neurons from *NEX-Shank3* WT (+/+) and KO (-/-) mice. **(d)** Input-output curves of NMDAR-EPSC and AMPAR-EPSC in response to a series of stimulation intensities in CA1 hippocampal neurons from *NEX-Shank3* WT (+/+) and KO (-/-) mice. All data are expressed as means ± SEM. For all panels, **p*<0.05, compared to wild-type controls.

# References

1. Wang X, Xu Q, Bey AL, Lee Y, Jiang YH. Transcriptional and functional complexity of Shank3 provides a molecular framework to understand the phenotypic heterogeneity of SHANK3 causing autism and Shank3 mutant mice. *Molecular autism* 2014; **5:** 30.

2. National Research Council. *Guide for the Care and Use of Laboratory Animals*, vol. 8th Edition: Washington (DC), 2011.

3. Wang X, Bey AL, Katz BM, Badea A, Kim N, David LK*, et al*. Altered mGluR5-Homer scaffolds and corticostriatal connectivity in a Shank3 complete knockout model of autism. *Nat Commun* 2016; **7:** 11459.

4. Rodriguiz RM, Chu R, Caron MG, Wetsel WC. Aberrant responses in social interaction of dopamine transporter knockout mice. *Behav Brain Res* 2004; **148**(1-2)**:** 185-198.

5. Wetsel WC, Rodriguiz RM, Guillemot J, Rousselet E, Essalmani R, Kim IH*, et al*. Disruption of the expression of the proprotein convertase PC7 reduces BDNF production and affects learning and memory in mice. *Proc Natl Acad Sci U S A* 2013; **110**(43)**:** 17362-17367.
